# Benchmarking Statistical, Machine Learning, and Exploratory Deep Learning Models for the Short-Term Forecasting of Monthly Aggregated Adult Ocular Surface Indicators: An Exploratory Hospital-Level Study

**DOI:** 10.3390/diagnostics16172856

**Published:** 2026-09-05

**Authors:** Ao Li, Ruijia Shi, Yanlin Wei, Yubo Wu, Jun Feng, Lei Tian, Ying Jie

**Affiliations:** 1 Beijing Institute of Ophthalmology, Beijing Tongren Eye Center, Beijing Tongren Hospital, Capital Medical University, Beijing Ophthalmology & Visual Sciences Key Laboratory, Beijing 100730, Chinashiruijia2004.0709@gmail.com (R.S.);; 2 School of Basic Medical Sciences, Capital Medical University, Beijing 100069, China

**Keywords:** non-invasive tear breakup time, tear meniscus height, ocular surface, time-series forecasting, hospital-level forecasting, benchmark study, rolling-origin validation

## Abstract

**Background/Objectives:** First NIBUT, Average NIBUT, and tear meniscus height (TMH) are routinely used to characterize tear film stability and tear volume at individual visits, whereas their longitudinal behavior across the hospital-attending population is less well characterized. Aggregating routine examinations over time may provide a continuous hospital-level view of ocular surface status and enable the short-term forecasting of expected trajectories. We therefore evaluated a benchmark-first framework for monthly aggregated adult ocular surface indicators. **Methods:** This retrospective time-series study used de-identified adult eye-level Keratograph 5M records from July 2018 to November 2023. After cleaning, 31,492 records from 13,749 patients and 15,334 examination occasions were aggregated across 65 calendar months (64 observed months; March 2020 had no eligible records). Seven benchmark models and two exploratory deep learning comparators were evaluated using eight rolling-origin 3-month test windows. **Results:** Linear trend had the lowest mean origin-level macro-normalized RMSE (0.875; 95% bootstrap CI, 0.497–1.421), followed by simple exponential smoothing (0.907) and SARIMA(1,0,0)(1,0,0,12) (0.940). The paired difference between linear trend and simple exponential smoothing was small and did not show clear superiority (mean difference, −0.032; 95% bootstrap CI, −0.217 to 0.135; *p* = 0.789). The model with the lowest pooled error differed by target, while patient-month and sample-size-weighted sensitivity analyses gave a similar overall benchmark pattern. The exploratory LSTM and Transformer did not show a consistent advantage over the leading simple models. **Conclusions:** In this short hospital-level monthly series, simple forecasting models remained competitive, while no single model showed consistent superiority across forecast origins and sensitivity analyses. By extending ocular surface assessment from isolated examinations to longitudinal hospital-level trajectories, this framework provides a methodological basis for monitoring temporal changes in tear film stability and tear volume and for future quality monitoring, clinical, and epidemiological applications.

## 1. Introduction

Dry eye disease and ocular surface dysfunction are common reasons for ophthalmic visits. The TFOS DEWS II reports describe dry eye as a multifactorial disease in which tear film homeostasis, ocular symptoms, inflammation, surface damage, and neurosensory abnormalities may all contribute to the clinical picture [1,2,3,4]. In routine clinical practice, however, a complete diagnostic dataset is not always available. Symptoms, corneal staining, Schirmer testing, meibomian gland grading, treatment history, and clinician-adjudicated diagnosis may be absent or unevenly recorded. By contrast, objective analyzer-based measurements are often generated repeatedly in large numbers.

Among these measurements, First NIBUT, Average NIBUT, and TMH have clear medical meaning. First NIBUT and Average NIBUT describe the stability of the tear film after blinking. They are related to the time-dependent breakdown of the tear film, which is central to visual fluctuation, ocular discomfort, and dry eye assessment. TMH reflects the lower tear meniscus and is commonly used as a non-invasive indicator of tear volume reserve. These variables should not be treated as a complete diagnosis, but they represent two important and complementary domains of ocular surface health: tear film stability and tear volume [2,5,6,7]. Monitoring them separately may therefore be more informative than merging all abnormalities into one broad label.

Current ocular surface assessment is largely centered on the patient’s status at a single clinical visit. In contrast, longitudinal changes in objective ocular surface indicators across the hospital-attending population remain less well characterized. Routine Keratograph examinations accumulate continuously in clinical practice and contain temporal information that can be transformed from isolated measurements into longitudinal hospital-level signals. Beyond describing ocular surface status at a single time point, longitudinal observation requires an understanding of how these indicators evolve over time and what short-term trajectory is expected. The continuous characterization and forecasting of hospital-level ocular surface indicators can provide a temporal reference for tracking changes in population patterns and for subsequent clinical and epidemiological investigation.

Real-world ocular surface data present several features that complicate longitudinal modeling, including bilateral measurements, repeated examinations, substantial variation in monthly sample size, indicator-specific missingness, heterogeneous temporal behavior across ocular surface measures, and relatively short time series. Monthly trends may also be influenced by patient mix, seasonal exposure, indoor environment, screen-related behavior, clinical workflow, and equipment or quality control factors. These factors were not included as explanatory covariates in the present forecasting models; the models therefore characterize the aggregate hospital-level temporal signal rather than attribute changes to a specific clinical or operational driver. These features make it important to establish a stable and reproducible forecasting framework before more complex models are considered. A benchmark-first design is especially relevant in medical AI, where added complexity should be judged against transparent and reproducible baselines.

The present study therefore aimed to extend ocular surface assessment from isolated clinical measurements to longitudinal hospital-level monitoring and short-term forecasting. We developed and systematically evaluated a benchmark-first framework for monthly aggregated First NIBUT, Average NIBUT, and TMH using routinely collected adult Keratograph 5M data. By comparing transparent statistical and machine learning benchmarks with exploratory deep learning models, we sought to identify a stable approach for characterizing the expected short-term trajectories of tear film stability and tear volume indicators in the hospital-attending population. This framework provides a methodological basis for longitudinal trend assessment, ocular surface care quality monitoring, and future clinical and epidemiological investigations of hospital-level ocular surface changes.

## 2. Materials and Methods

### 2.1. Study Design and Data Source

This was a retrospective hospital-level time-series benchmarking study. The source data came from de-identified clinical examination records obtained with the Keratograph 5M ocular surface analyzer (OCULUS Optikgeräte GmbH, Wetzlar, Germany) at Beijing Tongren Hospital, Capital Medical University, from July 2018 to November 2023. This study used historical analyzer/database records and did not involve new patient contact.

**Figure 1:**
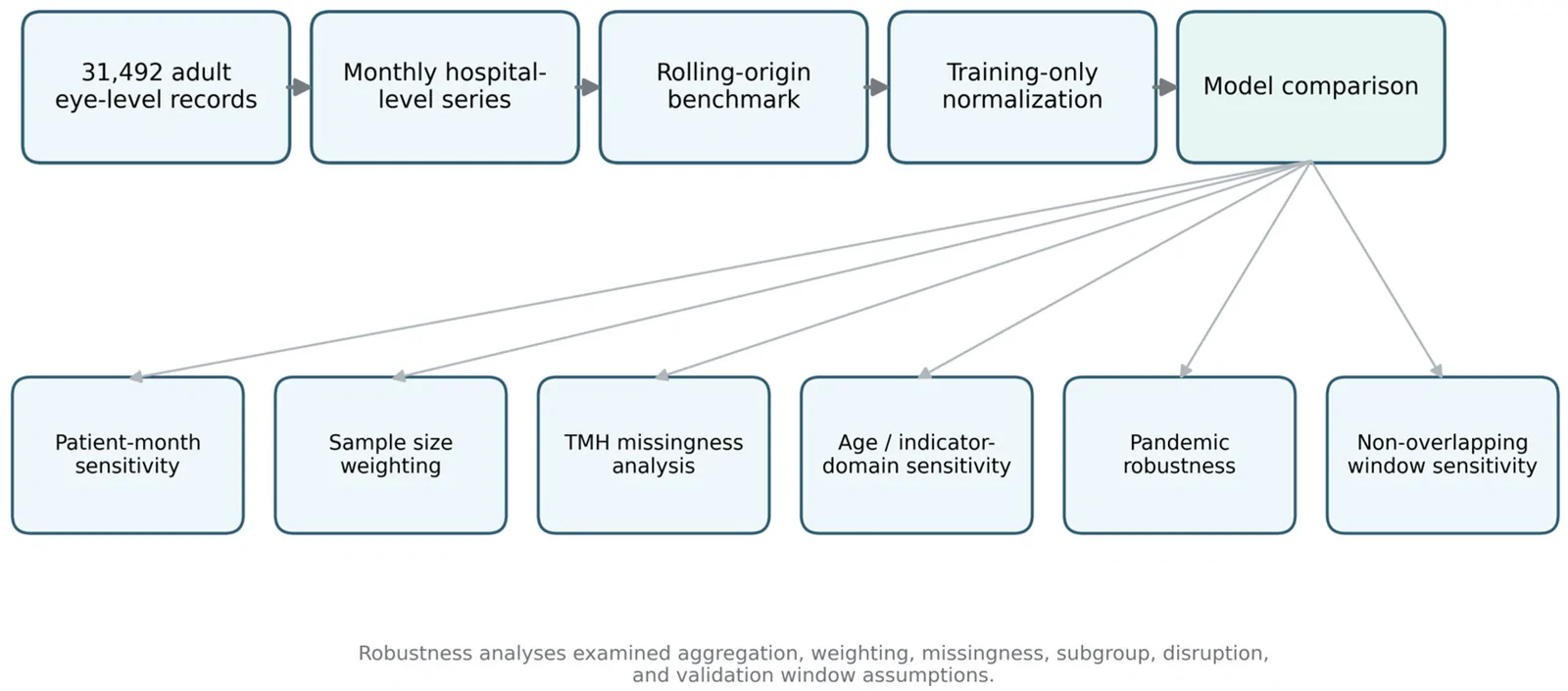
The study workflow and robustness analysis framework. The primary eye-level monthly series was evaluated with rolling-origin validation and training-only normalization; sensitivity analyses examined alternative aggregation and weighting, TMH missingness, age and indicator-domain definitions, pandemic period disruption, and overlapping forecast windows.

The analysis was approved by the Ethics Committee of Beijing Tongren Hospital, Capital Medical University (approval number: TREC2026-KYS139). Individual informed consent was waived because this study used de-identified historical clinical data. The analysis was restricted to adults aged 18 years or older. The original child and adolescent records were not used in the primary analysis. The overall study workflow and robustness analysis framework are summarized in Figure 1.

### 2.2. Adult Source Data and Monthly Aggregation

The cleaned adult source dataset contained 31,492 eye-level records from 13,749 unique patients and 15,334 examination occasions. Each record contained a de-identified patient identifier, a de-identified examination identifier, examination month, age, eye laterality, First NIBUT, Average NIBUT, and TMH. The analysis was restricted to adults aged ≥18 years. Eye laterality was standardized; nonnumeric target entries were treated as missing; and TMH values encoded in hundredths of a millimeter were divided by 100 and reported in millimeters. Repeated examinations were retained as part of the source hospital examination structure; no broad automatic duplicate removal or additional physiologic outlier trimming was applied.

**Figure 2:**
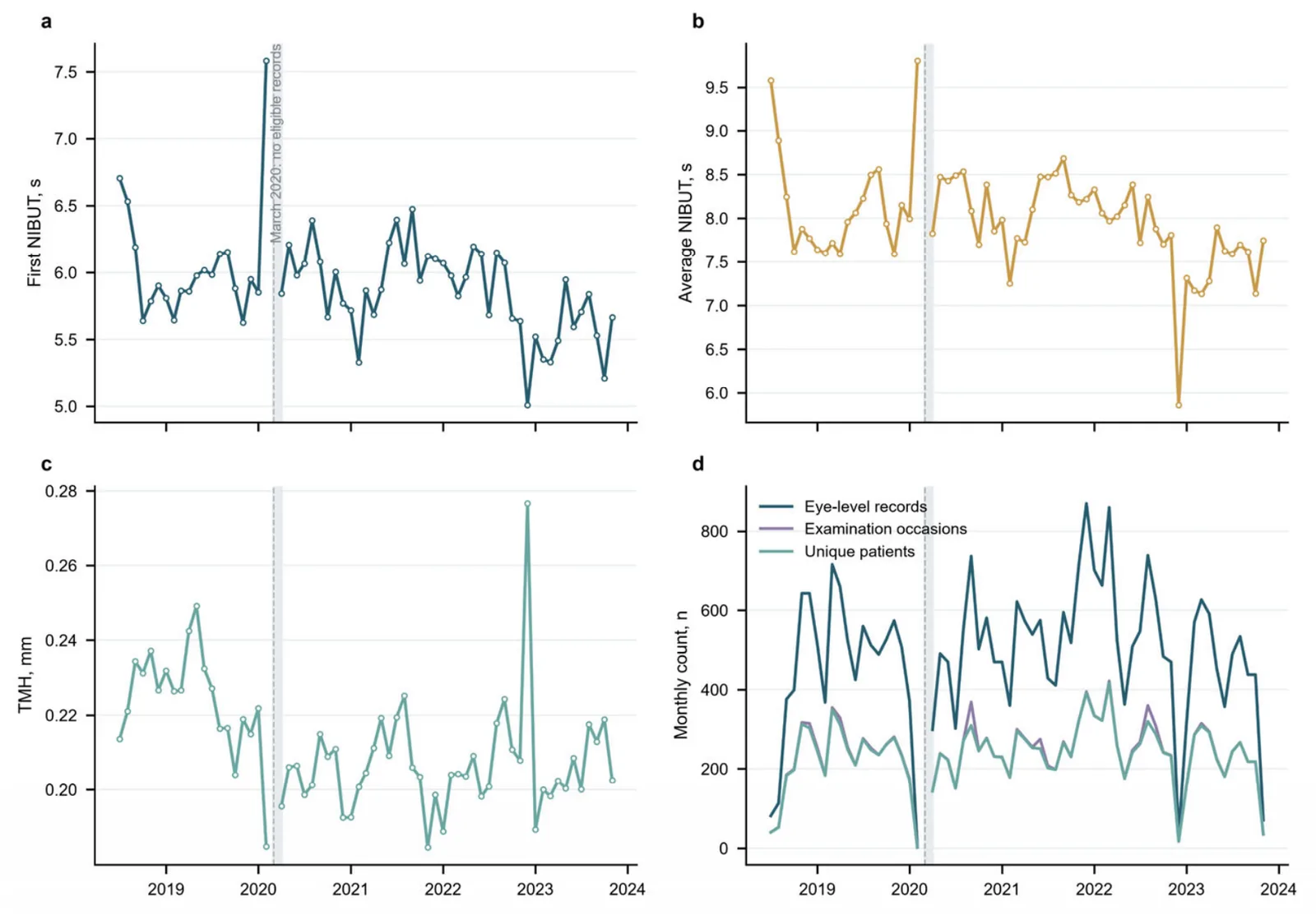
Monthly trends and the sample composition of adult ocular surface indicators. Panels show monthly (**a**) First NIBUT, (**b**) Average NIBUT, (**c**) TMH, and (**d**) the numbers of eye-level records, examination occasions, and unique patients across the study period. March 2020 is retained as a true observation gap; no interpolated value is displayed as observed.

The three forecasting targets were First NIBUT, Average NIBUT, and TMH. The primary analysis retained all eligible eye-level records and calculated calendar month means, representing the observed hospital-level analyzer signal rather than an equal-patient population estimate. For each observed month, the numbers of eye-level records, examination occasions, unique patients, and target-specific available and missing observations were summarized (Table S2). Observed trend plots were not interpolated. March 2020, the only month without eligible records, therefore appears as a gap in Figure 2.

The study period comprised 65 calendar months, of which 64 contained eligible observations. For each rolling-origin split, when March 2020 lay within the training series, the missing value for each target was linearly interpolated from February and April 2020; no interpolation was performed in any test window. Both months preceded every forecast origin, the earliest of which was December 2021; therefore, no validation period observation or test target was used for interpolation. The resulting model-ready series contained 65 calendar positions. First NIBUT and Average NIBUT were available for 31,467 of 31,492 records (99.92%). TMH was available for 22,950 records (72.88%) and missing for 8542 records (27.12%). The source-series characteristics and data completeness are summarized in Table 1.

### 2.3. Age-Stratified and Sensitivity Series

Age-stratified model-ready series were built for adults aged 18–35, 36–55, and ≥56 years. Because bilateral measurements and repeated examinations could yield unequal patient-level contributions to the primary eye-level monthly series, sensitivity analyses included examination-level averaging, right-eye-only series, and a patient-month series in which eye-level values were first averaged within examination and repeated examinations were then averaged within patient and month so that each patient contributed at most one equally weighted value per target per month.

Indicator-domain sensitivity analyses used operational definitions rather than clinical dry eye subtype classifications. Tear film instability was defined as a First NIBUT <10 s or Average NIBUT <10 s, consistent with commonly used non-invasive breakup time thresholds [2,8]. A stricter sensitivity definition used <5 s for either NIBUT measure; the 5 s threshold has been used as an abnormal First NIKBUT level in Keratograph-based assessment [9] and was applied here to both NIBUT measures as an operational sensitivity cutoff rather than a diagnostic severity category. Low tear volume was defined as a TMH <0.20 mm [8]. Combined definitions used the corresponding logical OR rules, with records remaining missing when no abnormal component was observed but at least one required component was unavailable (Table S3). TMH missingness was additionally summarized over calendar time, age strata, laterality, and monthly examination volume; Pearson and Spearman correlations were used to describe its association with monthly eye-level volume (Table S7). Device-, operator-, and room-level identifiers were unavailable, so these data collection factors could not be evaluated. As an exploratory pandemic period sensitivity analysis, segmented regressions used April 2020 as the post-disruption start and estimated level and slope terms; these analyses were descriptive and were not intended for causal inference (Table S6).

### 2.4. Forecasting Models and Validation

Seven benchmark models were evaluated: last-value naive, seasonal naive with a 12-month lag, simple exponential smoothing, ordinary least-squares linear trend, ARIMA(1,1,0), SARIMA(1,0,0)(1,0,0,12) with an intercept, and a random forest lag model. The statistical models were selected as common, interpretable baselines for short time series [10,11,12,13,14,15,16,17]. Simple exponential smoothing used an optimized smoothing parameter without a seasonal component. ARIMA and SARIMA orders were fixed across forecast origins to maintain a consistent benchmark specification in this short series. The random forest used six target lags, calendar month sine/cosine terms, 200 trees, maximum depth 4, minimum leaf size 1, max_features = 1.0, bootstrap sampling, and seed 42 [18,19].

A small LSTM and a lightweight Transformer were included as exploratory deep learning comparators [20,21]. Both used a 6-month lookback, training-only z-score scaling, full-batch optimization with AdamW (learning rate 0.001; weight decay 0.0001), mean squared error loss, a maximum of 500 epochs, an early-stopping patience of 50 epochs, and seed 42. The LSTM used one 16-unit recurrent layer with dropout 0.1; the Transformer used a model dimension of 16, one attention head, one encoder layer, a feed-forward dimension of 32, and dropout 0.1. Configurations were fixed across forecast origins without additional hyperparameter tuning. Full specifications are provided in Table S1.

Rolling-origin validation was used instead of a random split or single holdout. Training began in July 2018 for every split and expanded through the month immediately preceding each test window. Eight 3-month test windows were evaluated, January–March 2022, April–June 2022, July–September 2022, October–December 2022, January–March 2023, April–June 2023, July–September 2023, and August–October 2023, corresponding to forecast origins in December 2021, March 2022, June 2022, September 2022, December 2022, March 2023, June 2023, and July 2023, respectively. Forecast performance was evaluated separately at horizons h = 1, h = 2, and h = 3. The final two windows overlapped in calendar time and were not interpreted as statistically independent; two additional seven-window sensitivity sets removed either of these overlapping windows (Table S4).

### 2.5. Metrics and Ranking

Forecasts were evaluated on the original measurement scale. For each model, target, split, and analysis stratum, we calculated root mean square error (RMSE), mean absolute error, mean absolute percentage error, symmetric mean absolute percentage error, mean bias, and residual variability. Forecast accuracy metrics were selected to describe both average and scale-dependent error [15].

The primary ranking used macro-normalized RMSE. For each forecast origin and target, RMSE was divided by the standard deviation of that target in the corresponding training series only; no validation period observation contributed to the normalization factor. Target-specific normalized errors for First NIBUT, Average NIBUT, and TMH were then averaged. Origin-level macro-normalized RMSE values were used for 10,000-resample bootstrap 95% confidence intervals (seed 42), rank stability summaries, and exact paired sign flip comparisons among linear trend, simple exponential smoothing, and SARIMA. Non-significant paired comparisons were interpreted as no clear evidence of superiority, not equivalence. Sample-size-weighted RMSE and MAE used target-specific available counts as monthly weights; normalized weighted RMSE used the corresponding training period weighted standard deviation, and TMH weights used TMH-available rather than total eye-level counts (Table S6). Model-derived 95% prediction interval coverage for linear trend, ARIMA, and SARIMA was assessed as a supplementary uncertainty analysis (Table S5).

### 2.6. Software and Reproducibility

All analyses were performed in a staged Python 3.13 workflow that processed the adult eye-level data into monthly model-ready series and generated rolling-origin forecasts, performance tables, and figure source data. Time-series models were implemented with statsmodels [22], random forest and preprocessing with scikit-learn [19], and the exploratory deep learning models with PyTorch 2.13.0 [23].

## 3. Results

### 3.1. Adult Monthly Ocular Surface Indicator Series

The final adult source dataset included 31,492 eye-level records from 13,749 patients and 15,334 examination occasions across 65 calendar months from July 2018 to November 2023. Sixty-four months contained eligible observations; March 2020 contained no eligible records. First NIBUT and Average NIBUT were each available for 31,467 records (99.92%), whereas TMH was available for 22,950 records (72.88%). The monthly numbers of eye-level records, examination occasions, unique patients, and target-specific available and missing observations are provided in Table S2.

Across the 64 observed months, mean First NIBUT was 5.91 s (monthly standard deviation, 0.38; range, 5.01 to 7.58), mean Average NIBUT was 7.99 s (monthly standard deviation, 0.57; range, 5.86 to 9.80), and mean TMH was 0.212 mm (monthly standard deviation, 0.016; range, 0.185 to 0.277). TMH missingness varied over calendar time, ranging annually from 5.02% in 2019 to 57.23% in 2021 and declining to 8.65% in 2023. Missingness ranged from 25.76% to 29.14% across the three adult age strata and was similar between left and right eyes (27.28% and 26.97%, respectively). Univariable correlations between monthly TMH missingness and monthly eye-level volume were weak (Pearson r = 0.061, *p* = 0.633; Spearman ρ = 0.131, *p* = 0.303); detailed temporal and subgroup results are provided in Table S7.

### 3.2. Primary Overall Adult Model Benchmark

**Figure 3:**
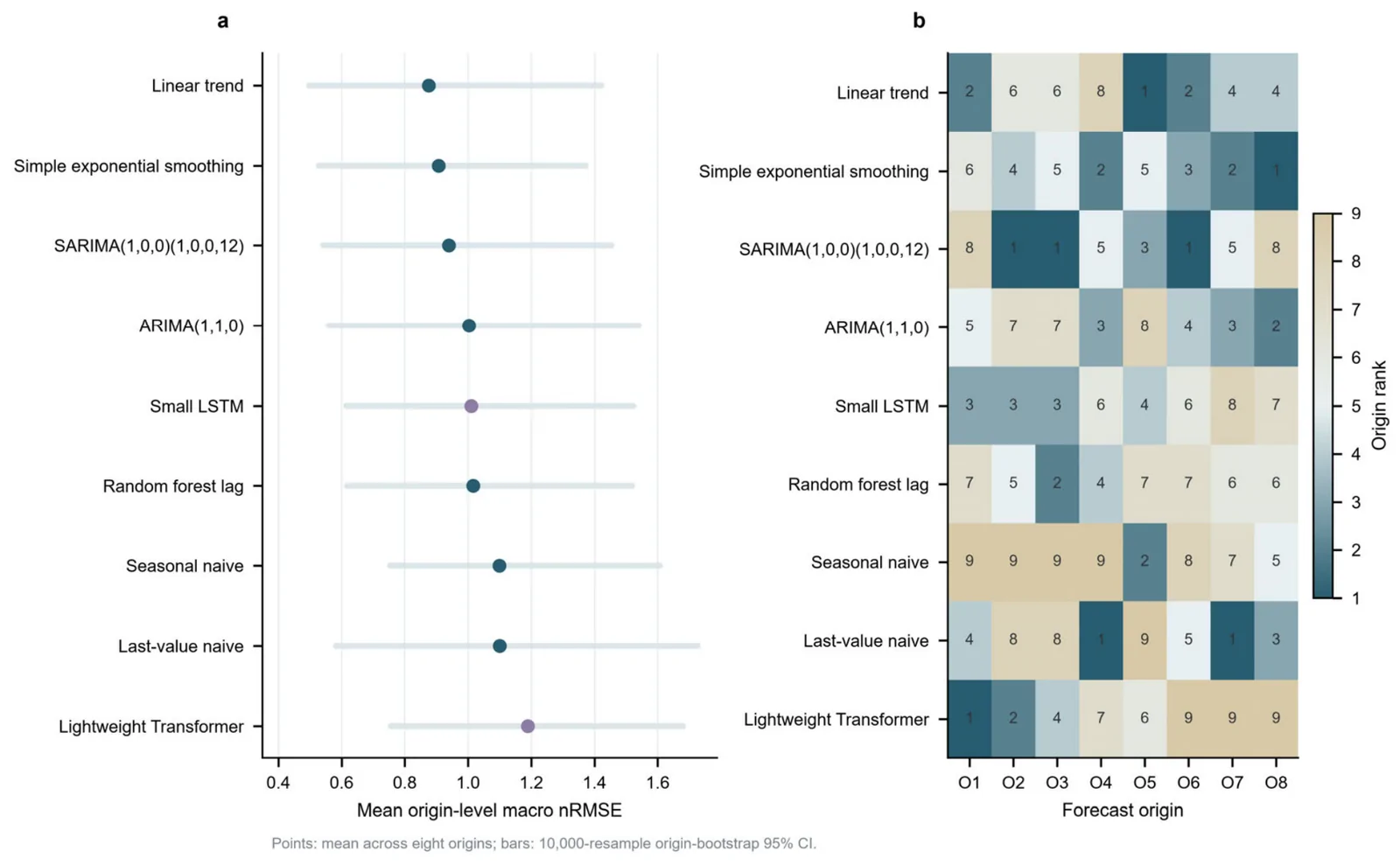
Primary benchmark performance and forecast-origin rank variability. (**a**) shows mean origin-level macro-normalized RMSE with 10,000-resample bootstrap 95% confidence intervals for all nine models. (**b**) shows origin-specific ranks; lower values indicate lower forecast error. No single model ranked first consistently.

In the primary overall adult benchmark, linear trend had the lowest point estimate of mean origin-level macro-normalized RMSE (0.875; 95% bootstrap CI, 0.497–1.421), followed by simple exponential smoothing (0.907) and SARIMA(1,0,0)(1,0,0,12) (0.940). Rankings varied across the eight forecast origins, and no model ranked first consistently (Table 2 and Table S4). The mean paired difference between linear trend and simple exponential smoothing was −0.032 (95% bootstrap CI, −0.217 to 0.135; exact sign-flip *p* = 0.789), providing no clear evidence of consistent superiority. Overall performance and forecast-origin rank variability are summarized in Table 2 and Figure 3.

The model with the lowest pooled RMSE differed by target: simple exponential smoothing for First NIBUT, linear trend for Average NIBUT, and seasonal naive for TMH. These target-specific differences were treated descriptively rather than as evidence of distinct biological forecasting mechanisms. Forecast-origin- and horizon-specific performance is reported in Tables S4 and S5.

### 3.3. Age-Stratified and Sensitivity Analyses

Point estimate rankings varied across adult age strata: simple exponential smoothing had the lowest macro-normalized RMSE among adults aged 18–35 years (0.884), SARIMA(1,0,0)(1,0,0,12) among those aged 36–55 years (0.914), and linear trend among those aged ≥56 years (0.928). Monthly composition, target availability, and complete model performance within each age stratum are provided in Table S8. These subgroup rankings were interpreted descriptively.

**Figure 4:**
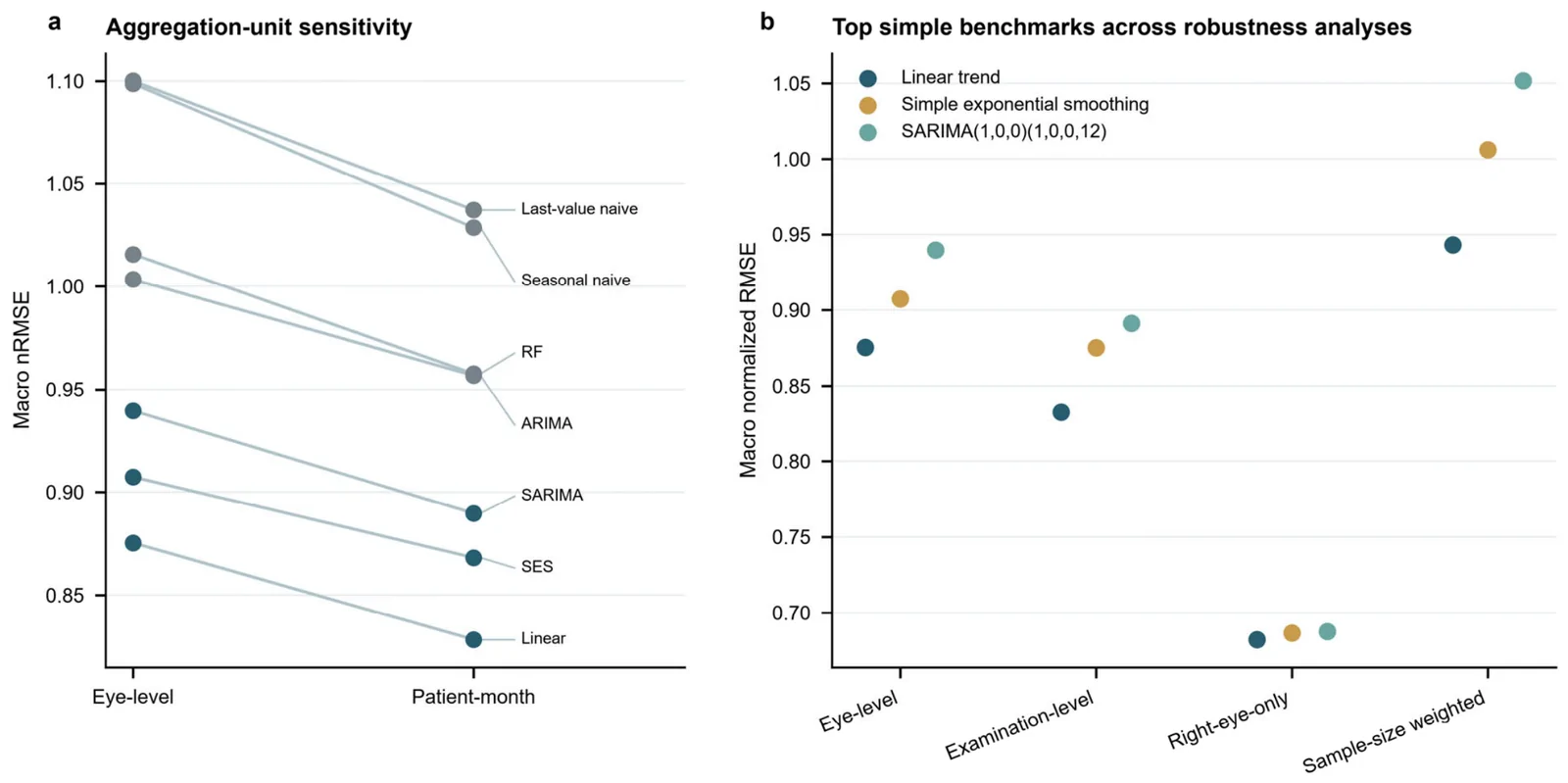
Robustness to aggregation and weighting choices. (**a**) compares eye-level and patient-month macro-normalized RMSE for the seven benchmark models. (**b**) shows the three leading simple benchmarks across eye-level, examination-level, right-eye-only, and sample-size-weighted analyses.

Sensitivity analyses gave a similar overall benchmark pattern under alternative aggregation and weighting choices. In the patient-month analysis, in which each patient contributed at most one equally weighted value per target per month, the ranking of the seven benchmark models was unchanged relative to the primary eye-level analysis (Spearman ρ = 1.00); macro-normalized RMSE was 0.828 for linear trend, 0.868 for simple exponential smoothing, and 0.890 for SARIMA (Table S6). In the sample-size-weighted analysis, the corresponding mean macro-normalized weighted RMSE values were 0.943, 1.006, and 1.052 (Table S6). Examination-level and right-eye-only analyses were consistent with this general pattern. Exploratory pandemic period analyses did not show clear evidence of an abrupt level change in First NIBUT (*p* = 0.955), Average NIBUT (*p* = 0.771), or TMH (*p* = 0.416) (Table S6), and the interpretation of no clear model superiority was unchanged when either overlapping final forecast window was removed (Table S4). These robustness analyses are summarized in Figure 4 and Table S6.

### 3.4. Exploratory Deep Learning Analysis

The small LSTM and lightweight Transformer were included as exploratory deep learning comparators, with 72 forecasts generated by each model. Their mean origin-level macro-normalized RMSE values were 1.010 and 1.189, respectively. Neither model showed a consistent forecasting advantage over the leading simple benchmarks (Table 2 and Tables S4 and S5).

Model-derived 95% prediction interval coverage for linear trend, ARIMA, and SARIMA varied by target and horizon; for TMH at h = 3, coverage was 75% for each of the three models (Table S5). These intervals were treated as a supplementary uncertainty assessment rather than evidence of established calibration.

## 4. Discussion

### 4.1. Main Findings

The principal contribution of this study is the demonstration that routinely collected ocular surface measurements can be transformed from isolated examination records into longitudinal hospital-level signals that support short-term forecasting. Linear trend had the lowest overall point estimate of macro-normalized RMSE, while simple exponential smoothing and SARIMA remained closely competitive. Rankings varied across forecast origins, targets, age strata, and sensitivity definitions, and no single model showed consistent superiority. The importance of these findings lies not only in model ranking but also in showing that short real-world ocular surface series can be used to construct an expected near-term trajectory at the hospital-service level.

This study was designed around a practical clinical services question: whether routine adult analyzer data can be converted into monthly hospital-level indicators with reproducible short-term trajectories. This framework adds a temporal dimension to routine ocular surface assessment and allows tear film stability and tear volume patterns to be considered evolving service-level signals rather than isolated measurements. Because these aggregate patterns may reflect patient composition, environmental exposure, clinical workflow, measurement practice, and other factors, their longitudinal characterization also creates a structured basis for investigating the determinants of hospital-level ocular surface change.

### 4.2. Medical Meaning of NIBUT and TMH Monitoring

NIBUT and TMH are not only convenient machine outputs. They represent different dimensions of ocular surface health. First NIBUT and Average NIBUT describe tear film stability after blinking. A shorter NIBUT is clinically relevant because an unstable tear film can cause intermittent blur, visual fatigue, ocular discomfort, and reduced quality of vision. It is also closely connected with the loss of tear film homeostasis described in TFOS reports [1,2,5]. TMH has a different meaning. It reflects the lower tear meniscus and provides a non-invasive estimate of tear volume reserve. Low TMH may indicate a reduced tear reservoir, while normal or high TMH does not necessarily imply normal tear film stability.

This separation is important for both medicine and modeling. If NIBUT and TMH are combined into a single abnormality label, the clinical message becomes blurred because they describe different measurement domains. The target-specific model rankings also differed descriptively; however, these differences may reflect variability, missingness, outliers, sampling structure, or temporal characteristics of the aggregated series and should not be interpreted as evidence of distinct biological forecasting mechanisms. The rationale for monitoring NIBUT and TMH separately therefore rests primarily on their complementary clinical measurement domains.

At the same time, these indicators must be interpreted with restraint. NIBUT and TMH alone cannot diagnose dry eye disease or separate aqueous-deficient from evaporative disease. An assessment of symptoms, staining, meibomian gland assessment, Schirmer testing, and clinician judgment remain necessary for individual-level diagnosis. The value of this study lies elsewhere. It shows that these two objective domains can be tracked as hospital-level indicators of ocular surface health, even when complete diagnostic variables are not available.

### 4.3. Clinical and Operational Relevance

Longitudinal forecasting adds a temporal dimension to routine ocular surface assessment. Monthly aggregation allows repeated clinical measurements to be interpreted not only as isolated observations but also as evolving hospital-level patterns of tear film stability and tear volume. Short-term forecasts provide an expected trajectory against which subsequent observations can be compared, creating a structured basis for tracking changes in the attending population. Similar forecasting concepts have been used in broader health-care capacity and service monitoring work [24], while ocular surface AI research has more often focused on tear film estimation or diagnostic classification [25,26]. The present study extends this work toward longitudinal hospital-level indicator forecasting.

Such longitudinal information may be particularly valuable in large ophthalmic services, where repeated ocular surface examinations accumulate continuously but are rarely analyzed as a coherent temporal signal. Converting these data into trackable and forecastable indicators provides an opportunity to integrate routine ocular surface measurements into broader quality monitoring and epidemiological research frameworks. At the aggregate monthly level, expected trajectories can support a structured review of changes in tear film stability and tear volume and provide a reference for closer clinical, measurement process, or population-level investigation. The present framework establishes expected trajectories rather than a prespecified alert or event detection rule, providing a foundation for the subsequent prospective evaluation of such functions.

Environmental conditions, digital device use, indoor air, air pollution, climate-related factors, patient attendance, and clinical workflow may influence ocular surface measurements [27,28,29,30]. Linking these factors with longitudinal hospital-level trajectories could help clarify how environmental exposures, seasonal variation, patient composition, measurement quality, and service-level changes relate to population ocular surface patterns over time. In this way, the forecasting framework provides a common temporal structure for future clinical, quality monitoring, and epidemiological analyses.

### 4.4. Why Simple Models Performed Well

The competitive performance of linear trend, simple exponential smoothing, and SARIMA is plausible in a short monthly series. Monthly aggregation reduces some individual-level variability and converts a clinical database into a low-frequency hospital-level signal. With only 65 calendar months, flexible models have limited information from which to learn stable nonlinear patterns, whereas simpler models require fewer parameters and provide transparent benchmarks [16,17].

Hospital-level ocular surface indicators may also evolve more smoothly than individual measurements because they summarize many examinations within each month. Such temporal structure can favor trend- or smoothing-based approaches. However, the present study was designed to compare forecasting performance rather than determine the mechanisms responsible for the observed temporal patterns.

### 4.5. Age and Sensitivity Analyses

Point estimate model rankings varied across adults aged 18–35, 36–55, and ≥56 years. This variation may reflect differences in monthly sample composition, target variability, missingness, or sampling uncertainty and should not be interpreted as evidence that different age groups have distinct underlying forecasting mechanisms. The age-stratified analyses therefore serve primarily as robustness assessments.

The sensitivity analyses were important because the primary dataset was eye-level. Patient-month, examination-level, right-eye-only, and sample-size-weighted analyses reduced concern that the main benchmark pattern was driven only by bilateral measurements, repeated examinations, or unequal monthly sample sizes. Indicator-domain analyses tested alternative operational definitions of tear film instability and low tear volume; these were robustness checks rather than clinical dry eye subtype cohorts.

### 4.6. Implications for Medical AI in Ocular Surface Disease

Neither exploratory deep learning model showed a consistent forecasting advantage over the leading simple benchmarks. In this short hospital-level monthly series, additional model complexity did not provide a stable performance advantage.

These findings support a benchmark-first approach when data are limited: additional model complexity should be evaluated against transparent baselines and justified by reproducible performance gains or clinically meaningful information. Longer series, external validation, richer covariates, and prospective evaluation would be needed to determine whether more complex models provide consistent advantages for hospital-level ocular surface forecasting.

### 4.7. Limitations

This study has several limitations. It was based on a single-center hospital dataset without external validation, and the forecasting series covered only 65 calendar months, limiting the precision with which small differences between models can be interpreted. The outcomes were monthly aggregated indicators rather than individual-level measurements, and the primary eye-level aggregation allowed for unequal contributions from patients with bilateral measurements or repeated examinations, although alternative aggregation and weighting analyses showed a similar overall benchmark pattern. March 2020 had no eligible records, and the study period included the COVID-19 pandemic, during which changes in attendance, case mix, or clinical workflow may have influenced the hospital-level signal. The source data also lacked complete diagnostic variables, and TMH had greater and temporally varying missingness than NIBUT. Environmental and clinic-workflow covariates were not included, and no validated operational threshold currently defines sufficient forecasting accuracy for monthly ocular surface surveillance. Future multicenter studies with longer series and external validation are needed.

## 5. Conclusions

In this 65-month hospital-level series, routinely collected First NIBUT, Average NIBUT, and TMH measurements could be transformed into longitudinal signals for short-term forecasting. Simple conventional forecasting models remained competitive; linear trend had the lowest overall point estimate of macro-normalized RMSE, but rankings varied across forecast origins and sensitivity analyses, and no model showed consistent superiority. First NIBUT, Average NIBUT, and TMH were retained as separate indicators to preserve their distinct clinical measurement domains, while exploratory LSTM and Transformer models did not demonstrate a stable performance advantage in this short series. By extending ocular surface assessment from isolated examinations to longitudinal hospital-level trajectories, this framework provides a practical methodological basis for monitoring temporal changes in tear film stability and tear volume and for future quality monitoring, clinical, and epidemiological applications.

## Figures and Tables

**Table 1:** Adult monthly ocular surface indicator series and data completeness.

Characteristic	Value
Adult eye-level records	31,492
Unique patients	13,749
Examination occasions	15,334
Study period	July 2018 to November 2023
Calendar months	65
Observed months	64
Missing calendar month	March 2020
Adult age strata	18–35, 36–55, and ≥56 years
First NIBUT availability	31,467/31,492 (99.92%)
Average NIBUT availability	31,467/31,492 (99.92%)
TMH availability/missingness	22,950/31,492 (72.88%); missing: 8542/31,492 (27.12%)
Sensitivity analyses	Patient-month, examination-level, right-eye-only, sample-size-weighted, age-stratified, indicator-domain, pandemic period, and non-overlapping window analyses

**Table 2:** Primary overall adult forecasting performance and rank stability.

Model	Mean Macro nRMSE	Bootstrap 95% CI	First-Rank *n*	Median Rank	Mean Rank
Linear trend	0.875	0.497–1.421	1	4	4.125
Simple exponential smoothing	0.907	0.528–1.372	1	3.500	3.500
SARIMA(1,0,0)(1,0,0,12)	0.940	0.542–1.454	3	4	4.000
ARIMA(1,1,0)	1.003	0.559–1.539	0	4.500	4.875
Small LSTM	1.010	0.615–1.523	0	5	5.000
Random forest lag	1.016	0.617–1.520	0	6	5.500
Seasonal naive	1.099	0.753–1.606	0	8.500	7.250
Last-value naive	1.100	0.582–1.726	2	4.500	4.875
Lightweight Transformer	1.189	0.755–1.681	1	6.500	5.875

## Data Availability

The minimal dataset cannot be publicly uploaded because this study was based on de-identified historical hospital clinical examination records. Although direct identifiers were removed, the underlying source data remain subject to patient privacy protection, institutional data governance policies, and ethics approval restrictions. The public deposition of patient-level or visit-level clinical data was not approved. The main aggregated results, model performance summaries, and figure-level data are included in this manuscript and Supplementary Materials. De-identified aggregated monthly data and analysis code may be made available from the corresponding author upon reasonable request, subject to institutional approval and applicable ethics and data use requirements.

## Supplementary Materials

The following supporting information can be downloaded at https://www.mdpi.com/article/10.3390/diagnostics16172856/s1, Table S1. Model specifications. Table S2. Adult source dataset and monthly sample composition. Table S3. Exact indicator-domain definitions, thresholds, denominators, and missing-data rules. Table S4. Forecast-origin performance, paired comparisons, and non-overlapping-window sensitivity. Table S5. Horizon-specific performance and prediction-interval coverage. Table S6. Robustness analyses: aggregation, sample-size weighting, and pandemic-period sensitivity. Table S7. TMH missingness analysis. Table S8. Age-stratified data composition and model performance.
