# Supplementary material for: Benchmarking Statistical, Machine Learning, and Exploratory Deep Learning Models for the Short-Term Forecasting of Monthly Aggregated Adult Ocular Surface Indicators: An Exploratory Hospital-Level Study
Source: Diagnostics (Basel). 2026 Sep 5;16(17):2856. doi: 10.3390/diagnostics16172856 (PMC13565034; doi:10.3390/diagnostics16172856)
Supplement: Supplementary file 1 [file diagnostics-16-02856-s001.zip › diagnostics-4444403-supplementary.pdf]

Supplementary Materials

Benchmarking Statistical, Machine-Learning, and Exploratory Deep-Learning Models for Short-Term Forecasting of Monthly Aggregated Adult Ocular Surface Indicators: An Exploratory Hospital-Level Study

This supplement provides Tables S1–S8. NIBUT is reported in seconds and TMH in millimeters. Abbreviations: NIBUT, non-invasive tear break-up time; TMH, tear meniscus height; RMSE, root mean square error; nRMSE, normalized root mean square error; MAE, mean absolute error; CI, confidence interval; PI, prediction interval; LSTM, long short-term memory; RF, random forest.

Table S1. Model specifications

Model	Model class/specification	Key parameters	Role in comparison
Last-value naive	Naive benchmark	Most recent observed training value	Benchmark comparator
Seasonal naive	Seasonal naive benchmark	12-month calendar lag	Benchmark comparator
Simple exponential smoothing	Exponential-smoothing benchmark	Optimized smoothing; no seasonal component	Benchmark comparator
Linear trend	Ordinary least-squares time trend	Calendar-time predictor	Benchmark comparator
ARIMA(1,1,0)	Fixed-order ARIMA	Order (1,1,0)	Benchmark comparator with model-derived prediction intervals
SARIMA(1,0,0)(1,0,0,12)	Fixed-order seasonal ARIMA	Order (1,0,0)(1,0,0,12) with intercept	Benchmark comparator with model-derived prediction intervals
Random forest lag	Tree-based lag model	Six target lags; month sine/cosine; 200 trees; depth 4; minimum leaf 1; max_features 1.0; bootstrap; seed 42	Machine-learning benchmark comparator
Small LSTM	Exploratory recurrent neural network	Lookback 6; one LSTM layer with 16 hidden units; dropout 0.1; training-only z-score; full-batch; AdamW (lr 0.001; weight decay 0.0001); ≤500 epochs; patience 50; seed 42	Exploratory deep-learning comparator
Lightweight Transformer	Exploratory attention model	Lookback 6; d_model 16; one head and one layer; feed-forward dimension 32; dropout 0.1; training-only z-score; full-batch; AdamW (lr 0.001; weight decay 0.0001); ≤500 epochs; patience 50; seed 42	Exploratory deep-learning comparator

Table S2. Adult source dataset and monthly sample composition

A. Source composition

Characteristic	Value	Definition
Adult eye-level records	31,492	All eligible adult eye-level rows
Unique patients	13,749	Distinct de-identified patient identifiers
Examination occasions	15,334	Distinct de-identified examination identifiers
Study period	July 2018–November 2023	Retrospective hospital-level series

Characteristic	Value	Definition
Calendar months	65	Inclusive calendar span
Observed months	64	Months with at least one eligible record
Missing calendar month	March 2020	No eligible records
First NIBUT available	31,467 (99.92%)	Non-missing eye-level values
Average NIBUT available	31,467 (99.92%)	Non-missing eye-level values
TMH available	22,950 (72.88%)	Non-missing eye-level values
TMH missingness	8,542 (27.12%)	Missing eye-level values
Median monthly eye-level records (IQR)	510 (422–584)	Across 64 observed months
Median monthly unique patients (IQR)	244 (201–281)	Across 64 observed months
Median monthly examination occasions (IQR)	246 (206–282)	Across 64 observed months

B. Monthly composition (overall)

Calendar month	Age stratum	Eye-level records	Unique examinations	Unique patients	Examinations per patient	Eye records per patient	First NIBUT available	Average NIBUT available	TMH available	TMH missing	TMH missing (%)
2018-07	Overall	81	40	40	1.000	2.025	81	81	22	59	72.84
2018-08	Overall	113	53	52	1.019	2.173	113	113	19	94	83.19
2018-09	Overall	376	185	183	1.011	2.055	376	376	333	43	11.44
2018-10	Overall	399	199	197	1.010	2.025	399	399	360	39	9.77
2018-11	Overall	643	317	313	1.013	2.054	643	643	604	39	6.07
2018-12	Overall	643	315	303	1.040	2.122	641	641	564	79	12.29
2019-01	Overall	515	253	245	1.033	2.102	514	514	473	42	8.16
2019-02	Overall	368	184	183	1.005	2.011	368	368	339	29	7.88
2019-03	Overall	716	355	350	1.014	2.046	714	714	642	74	10.34
2019-04	Overall	660	329	312	1.054	2.115	659	659	609	51	7.73
2019-05	Overall	524	255	249	1.024	2.104	524	524	502	22	4.20
2019-06	Overall	425	209	209	1.000	2.033	425	425	408	17	4.00
2019-07	Overall	560	277	275	1.007	2.036	558	558	543	17	3.04
2019-08	Overall	512	253	248	1.020	2.065	512	512	498	14	2.73
2019-09	Overall	489	235	235	1.000	2.081	487	487	481	8	1.64
2019-10	Overall	526	262	261	1.004	2.015	526	526	511	15	2.85
2019-11	Overall	574	281	279	1.007	2.057	573	573	563	11	1.92

Calendar month	Age stratum	Eye-level records	Unique examinations	Unique patients	Examinations per patient	Eye records per patient	First NIBUT available	Average NIBUT available	TMH available	TMH missing	TMH missing (%)
2019-12	Overall	507	235	233	1.009	2.176	505	505	487	20	3.94
2020-01	Overall	371	174	171	1.018	2.170	371	371	360	11	2.96
2020-02	Overall	4	2	2	1.000	2.000	4	4	4	0	0.00
2020-03	Overall	0	0	0			0	0	0	0	
2020-04	Overall	299	143	143	1.000	2.091	299	299	291	8	2.68
2020-05	Overall	491	239	239	1.000	2.054	490	490	471	20	4.07
2020-06	Overall	470	223	222	1.005	2.117	470	470	454	16	3.40
2020-07	Overall	302	150	150	1.000	2.013	301	301	296	6	1.99
2020-08	Overall	551	271	271	1.000	2.033	551	551	536	15	2.72
2020-09	Overall	737	369	310	1.190	2.377	734	734	672	65	8.82
2020-10	Overall	502	246	245	1.004	2.049	502	502	232	270	53.78
2020-11	Overall	581	278	278	1.000	2.090	581	581	227	354	60.93
2020-12	Overall	470	231	231	1.000	2.035	470	470	228	242	51.49
2021-01	Overall	470	230	229	1.004	2.052	470	470	198	272	57.87
2021-02	Overall	360	178	178	1.000	2.022	360	360	140	220	61.11
2021-03	Overall	622	300	296	1.014	2.101	622	622	275	347	55.79
2021-04	Overall	573	277	275	1.007	2.084	573	573	247	326	56.89
2021-05	Overall	539	255	253	1.008	2.130	539	539	255	284	52.69
2021-06	Overall	575	275	251	1.096	2.291	575	575	276	299	52.00
2021-07	Overall	429	208	202	1.030	2.124	429	429	146	283	65.97
2021-08	Overall	411	198	198	1.000	2.076	411	411	166	245	59.61
2021-09	Overall	595	269	267	1.007	2.228	595	595	209	386	64.87
2021-10	Overall	518	231	230	1.004	2.252	518	518	219	299	57.72
2021-11	Overall	715	322	322	1.000	2.220	714	714	296	419	58.60
2021-12	Overall	871	395	392	1.008	2.222	871	871	429	442	50.75
2022-01	Overall	702	335	334	1.003	2.102	702	702	321	381	54.27
2022-02	Overall	663	322	322	1.000	2.059	663	663	300	363	54.75
2022-03	Overall	861	422	417	1.012	2.065	861	861	523	338	39.26
2022-04	Overall	525	260	259	1.004	2.027	525	525	251	274	52.19
2022-05	Overall	363	177	175	1.011	2.074	363	363	132	231	63.64

Calendar month	Age stratum	Eye-level records	Unique examinations	Unique patients	Examinations per patient	Eye records per patient	First NIBUT available	Average NIBUT available	TMH available	TMH missing	TMH missing (%)
2022-06	Overall	509	247	242	1.021	2.103	508	508	204	305	59.92
2022-07	Overall	547	268	263	1.019	2.080	547	547	350	197	36.01
2022-08	Overall	739	360	320	1.125	2.309	739	739	523	216	29.23
2022-09	Overall	629	309	288	1.073	2.184	629	629	430	199	31.64
2022-10	Overall	484	242	241	1.004	2.008	484	484	437	47	9.71
2022-11	Overall	470	234	233	1.004	2.017	470	470	411	59	12.55
2022-12	Overall	32	17	17	1.000	1.882	32	32	24	8	25.00
2023-01	Overall	315	158	156	1.013	2.019	314	314	273	42	13.33
2023-02	Overall	570	286	286	1.000	1.993	570	570	494	76	13.33
2023-03	Overall	627	315	309	1.019	2.029	627	627	577	50	7.97
2023-04	Overall	591	294	292	1.007	2.024	590	590	539	52	8.80
2023-05	Overall	452	225	223	1.009	2.027	452	452	395	57	12.61
2023-06	Overall	357	180	180	1.000	1.983	357	357	327	30	8.40
2023-07	Overall	489	244	243	1.004	2.012	488	488	463	26	5.32
2023-08	Overall	534	267	267	1.000	2.000	534	534	501	33	6.18
2023-09	Overall	438	218	218	1.000	2.009	438	438	406	32	7.31
2023-10	Overall	438	218	218	1.000	2.009	436	436	417	21	4.79
2023-11	Overall	70	35	35	1.000	2.000	70	70	67	3	4.29

Table S3. Exact indicator-domain definitions, thresholds, denominators, and missing-data rules

Indicator definitions

Indicator	Exact definition	Threshold	Operator	Required measurements	Denominator	Missing-data rule
Tear-film instability	First NIBUT <10 OR Average NIBUT <10	10 s	<	First and Average NIBUT	Records with non-missing derived classification	Positive if either observed NIBUT measure met the threshold; negative only if both required NIBUT measures were observed and normal; otherwise missing
Strict tear-film instability	First NIBUT <5 OR Average NIBUT <5	5 s	<	First and Average NIBUT	Records with non-missing derived classification	Positive if either observed NIBUT measure met the threshold; negative only if both required NIBUT measures were observed and normal; otherwise missing
Low tear volume	TMH <0.20 mm	0.20 mm	<	TMH	Records with observed TMH	Missing TMH remained missing

Indicator	Exact definition	Threshold	Operator	Required measurements	Denominator	Missing-data rule
Combined indicator abnormality	First NIBUT <10 OR Average NIBUT <10 OR TMH <0.20	10 s / 0.20 mm	<	First NIBUT, Average NIBUT, and TMH	Records with non-missing derived classification	Positive if any observed component was abnormal; negative only if all required components were observed and normal; otherwise missing
Strict combined indicator abnormality	First NIBUT <5 OR Average NIBUT <5 OR TMH <0.20	5 s / 0.20 mm	<	First NIBUT, Average NIBUT, and TMH	Records with non-missing derived classification	Same three-state rule as the combined indicator

Table S4. Forecast-origin performance, paired comparisons, and non-overlapping-window sensitivity

A. Primary origin-level performance

Dataset	Model	Origin no.	Macro nRMSE	Rank
Eye-level primary	ARIMA(1,1,0)	1	0.439	5
Eye-level primary	ARIMA(1,1,0)	2	0.503	7
Eye-level primary	ARIMA(1,1,0)	3	0.947	7
Eye-level primary	ARIMA(1,1,0)	4	2.285	3
Eye-level primary	ARIMA(1,1,0)	5	2.086	8
Eye-level primary	ARIMA(1,1,0)	6	0.754	4
Eye-level primary	ARIMA(1,1,0)	7	0.324	3
Eye-level primary	ARIMA(1,1,0)	8	0.689	2
Eye-level primary	Last-value naive	1	0.430	4
Eye-level primary	Last-value naive	2	0.540	8
Eye-level primary	Last-value naive	3	1.004	8
Eye-level primary	Last-value naive	4	2.229	1
Eye-level primary	Last-value naive	5	2.753	9
Eye-level primary	Last-value naive	6	0.772	5
Eye-level primary	Last-value naive	7	0.315	1
Eye-level primary	Last-value naive	8	0.758	3
Eye-level primary	Lightweight Transformer	1	0.362	1
Eye-level primary	Lightweight Transformer	2	0.394	2
Eye-level primary	Lightweight Transformer	3	0.843	4
Eye-level primary	Lightweight Transformer	4	2.567	7

Dataset	Model	Origin no.	Macro nRMSE	Rank
Eye-level primary	Lightweight Transformer	5	1.722	6
Eye-level primary	Lightweight Transformer	6	1.160	9
Eye-level primary	Lightweight Transformer	7	1.118	9
Eye-level primary	Lightweight Transformer	8	1.341	9
Eye-level primary	Linear trend	1	0.398	2
Eye-level primary	Linear trend	2	0.434	6
Eye-level primary	Linear trend	3	0.908	6
Eye-level primary	Linear trend	4	2.621	8
Eye-level primary	Linear trend	5	1.025	1
Eye-level primary	Linear trend	6	0.442	2
Eye-level primary	Linear trend	7	0.395	4
Eye-level primary	Linear trend	8	0.779	4
Eye-level primary	Small LSTM	1	0.428	3
Eye-level primary	Small LSTM	2	0.402	3
Eye-level primary	Small LSTM	3	0.773	3
Eye-level primary	Small LSTM	4	2.566	6
Eye-level primary	Small LSTM	5	1.536	4
Eye-level primary	Small LSTM	6	0.830	6
Eye-level primary	Small LSTM	7	0.674	8
Eye-level primary	Small LSTM	8	0.869	7
Eye-level primary	Random forest lag	1	0.515	7
Eye-level primary	Random forest lag	2	0.413	5
Eye-level primary	Random forest lag	3	0.767	2
Eye-level primary	Random forest lag	4	2.468	4
Eye-level primary	Random forest lag	5	1.734	7
Eye-level primary	Random forest lag	6	0.835	7
Eye-level primary	Random forest lag	7	0.561	6
Eye-level primary	Random forest lag	8	0.833	6
Eye-level primary	SARIMA(1,0,0)(1,0,0,12)	1	0.571	8

Dataset	Model	Origin no.	Macro nRMSE	Rank
Eye-level primary	SARIMA(1,0,0)(1,0,0,12)	2	0.332	1
Eye-level primary	SARIMA(1,0,0)(1,0,0,12)	3	0.730	1
Eye-level primary	SARIMA(1,0,0)(1,0,0,12)	4	2.517	5
Eye-level primary	SARIMA(1,0,0)(1,0,0,12)	5	1.452	3
Eye-level primary	SARIMA(1,0,0)(1,0,0,12)	6	0.422	1
Eye-level primary	SARIMA(1,0,0)(1,0,0,12)	7	0.555	5
Eye-level primary	SARIMA(1,0,0)(1,0,0,12)	8	0.937	8
Eye-level primary	Seasonal naive	1	0.775	9
Eye-level primary	Seasonal naive	2	0.556	9
Eye-level primary	Seasonal naive	3	1.241	9
Eye-level primary	Seasonal naive	4	2.730	9
Eye-level primary	Seasonal naive	5	1.125	2
Eye-level primary	Seasonal naive	6	0.892	8
Eye-level primary	Seasonal naive	7	0.649	7
Eye-level primary	Seasonal naive	8	0.823	5
Eye-level primary	Simple exponential smoothing	1	0.453	6
Eye-level primary	Simple exponential smoothing	2	0.406	4
Eye-level primary	Simple exponential smoothing	3	0.877	5
Eye-level primary	Simple exponential smoothing	4	2.255	2
Eye-level primary	Simple exponential smoothing	5	1.549	5
Eye-level primary	Simple exponential smoothing	6	0.736	3
Eye-level primary	Simple exponential smoothing	7	0.322	2
Eye-level primary	Simple exponential smoothing	8	0.661	1

B. Primary paired comparisons

Comparison	Mean paired difference in macro nRMSE	95% bootstrap CI	Exact sign-flip P	Origins favoring model A	Origins favoring model B	Interpretation
Linear trend vs Simple exponential smoothing	−0.032	−0.217 to 0.135	0.789	3	5	No clear evidence of superiority
Linear trend vs SARIMA(1,0,0)(1,0,0,12)	−0.064	−0.198 to 0.064	0.406	4	4	No clear evidence of superiority

Comparison	Mean paired difference in macro nRMSE	95% bootstrap CI	Exact sign-flip P	Origins favoring model A	Origins favoring model B	Interpretation
Simple exponential smoothing vs SARIMA(1,0,0)(1,0,0,12)	−0.032	−0.169 to 0.115	0.680	4	4	No clear evidence of superiority

C. Non-overlapping-window paired comparisons

Origin set	Comparison	Mean paired difference in macro nRMSE	95% bootstrap CI lower	95% bootstrap CI upper	Exact sign-flip P	Origins favoring model A	Origins favoring model B	Ties	Interpretation
Set A: origins 1–7	Linear trend vs Simple exponential smoothing	−0.053	−0.254	0.132	0.703	3	4	0	No clear evidence of superiority was observed.
Set A: origins 1–7	Linear trend vs SARIMA(1,0,0)(1,0,0,12)	−0.051	−0.204	0.085	0.594	3	4	0	No clear evidence of superiority was observed.
Set A: origins 1–7	Simple exponential smoothing vs SARIMA(1,0,0)(1,0,0,12)	0.003	−0.140	0.149	0.984	3	4	0	No clear evidence of superiority was observed.
Set B: origins 1–6 and 8	Linear trend vs Simple exponential smoothing	−0.047	−0.253	0.150	0.719	3	4	0	No clear evidence of superiority was observed.
Set B: origins 1–6 and 8	Linear trend vs SARIMA(1,0,0)(1,0,0,12)	−0.051	−0.204	0.085	0.594	3	4	0	No clear evidence of superiority was observed.
Set B: origins 1–6 and 8	Simple exponential smoothing vs SARIMA(1,0,0)(1,0,0,12)	−0.004	−0.153	0.146	0.969	3	4	0	No clear evidence of superiority was observed.

Table S5. Horizon-specific performance and prediction-interval coverage

A. Primary horizon-specific performance

Dataset	Model	Horizon	Target	Forecasts	RMSE	MAE	MAPE	sMAPE	Bias	Normalized RMSE
Eye-level primary	ARIMA(1,1,0)	1	Average NIBUT	8	0.423	0.293	3.889	3.961	−0.041	0.828
Eye-level primary	ARIMA(1,1,0)	1	First NIBUT	8	0.272	0.214	3.779	3.738	0.023	0.718
Eye-level primary	ARIMA(1,1,0)	1	TMH	8	0.026	0.014	7.145	6.411	0.010	1.676
Eye-level primary	ARIMA(1,1,0)	2	Average NIBUT	8	0.439	0.286	3.763	3.936	−0.187	0.859
Eye-level primary	ARIMA(1,1,0)	2	First NIBUT	8	0.318	0.259	4.462	4.521	−0.076	0.839
Eye-level primary	ARIMA(1,1,0)	2	TMH	8	0.025	0.017	8.059	7.563	0.003	1.610
Eye-level primary	ARIMA(1,1,0)	3	Average NIBUT	8	0.869	0.639	9.458	8.867	0.199	1.701
Eye-level primary	ARIMA(1,1,0)	3	First NIBUT	8	0.458	0.348	6.513	6.216	0.167	1.209
Eye-level primary	ARIMA(1,1,0)	3	TMH	8	0.032	0.024	10.599	10.421	−0.006	2.059
Eye-level primary	Last-value naive	1	Average NIBUT	8	0.575	0.343	4.579	4.812	−0.125	1.125

Dataset	Model	Horizon	Target	Forecasts	RMSE	MAE	MAPE	sMAPE	Bias	Normalized RMSE
Eye-level primary	Last-value naive	1	First NIBUT	8	0.301	0.245	4.341	4.340	−0.019	0.793
Eye-level primary	Last-value naive	1	TMH	8	0.032	0.018	9.191	8.107	0.012	2.082
Eye-level primary	Last-value naive	2	Average NIBUT	8	0.548	0.341	4.513	4.805	−0.248	1.072
Eye-level primary	Last-value naive	2	First NIBUT	8	0.341	0.290	5.009	5.095	−0.106	0.899
Eye-level primary	Last-value naive	2	TMH	8	0.029	0.018	8.922	8.209	0.005	1.890
Eye-level primary	Last-value naive	3	Average NIBUT	8	0.912	0.679	9.986	9.559	0.132	1.785
Eye-level primary	Last-value naive	3	First NIBUT	8	0.466	0.359	6.713	6.443	0.133	1.230
Eye-level primary	Last-value naive	3	TMH	8	0.036	0.025	11.426	11.013	−0.004	2.298
Eye-level primary	Lightweight Transformer	1	Average NIBUT	8	0.453	0.407	5.337	5.173	0.356	0.887
Eye-level primary	Lightweight Transformer	1	First NIBUT	8	0.243	0.202	3.573	3.488	0.150	0.640
Eye-level primary	Lightweight Transformer	1	TMH	8	0.019	0.014	6.963	6.521	0.013	1.226
Eye-level primary	Lightweight Transformer	2	Average NIBUT	8	0.521	0.419	5.523	5.319	0.318	1.019
Eye-level primary	Lightweight Transformer	2	First NIBUT	8	0.444	0.375	6.626	6.383	0.204	1.170
Eye-level primary	Lightweight Transformer	2	TMH	8	0.016	0.011	5.540	5.305	0.005	1.032
Eye-level primary	Lightweight Transformer	3	Average NIBUT	8	1.116	0.904	13.158	11.872	0.850	2.184
Eye-level primary	Lightweight Transformer	3	First NIBUT	8	0.693	0.559	10.514	9.718	0.547	1.827
Eye-level primary	Lightweight Transformer	3	TMH	8	0.030	0.021	9.052	9.510	−0.007	1.915
Eye-level primary	Linear trend	1	Average NIBUT	8	0.357	0.296	3.909	3.802	0.257	0.699
Eye-level primary	Linear trend	1	First NIBUT	8	0.242	0.196	3.477	3.392	0.156	0.639
Eye-level primary	Linear trend	1	TMH	8	0.010	0.008	4.184	4.216	−0.003	0.660
Eye-level primary	Linear trend	2	Average NIBUT	8	0.293	0.189	2.504	2.433	0.129	0.574
Eye-level primary	Linear trend	2	First NIBUT	8	0.262	0.218	3.835	3.760	0.066	0.692
Eye-level primary	Linear trend	2	TMH	8	0.013	0.011	5.266	5.457	−0.010	0.849
Eye-level primary	Linear trend	3	Average NIBUT	8	0.881	0.554	8.470	7.571	0.504	1.724
Eye-level primary	Linear trend	3	First NIBUT	8	0.457	0.348	6.578	6.218	0.301	1.206
Eye-level primary	Linear trend	3	TMH	8	0.032	0.021	8.719	9.542	−0.020	2.044
Eye-level primary	Small LSTM	1	Average NIBUT	8	0.496	0.472	6.193	6.001	0.397	0.970
Eye-level primary	Small LSTM	1	First NIBUT	8	0.331	0.285	5.068	4.902	0.262	0.872
Eye-level primary	Small LSTM	1	TMH	8	0.013	0.011	5.824	5.616	0.008	0.872
Eye-level primary	Small LSTM	2	Average NIBUT	8	0.349	0.223	2.986	2.877	0.207	0.683

Dataset	Model	Horizon	Target	Forecasts	RMSE	MAE	MAPE	sMAPE	Bias	Normalized RMSE
Eye-level primary	Small LSTM	2	First NIBUT	8	0.310	0.213	3.838	3.688	0.165	0.818
Eye-level primary	Small LSTM	2	TMH	8	0.009	0.007	3.354	3.330	−0.000	0.576
Eye-level primary	Small LSTM	3	Average NIBUT	8	0.985	0.726	10.820	9.747	0.678	1.927
Eye-level primary	Small LSTM	3	First NIBUT	8	0.595	0.493	9.257	8.661	0.453	1.571
Eye-level primary	Small LSTM	3	TMH	8	0.029	0.017	6.799	7.329	−0.009	1.863
Eye-level primary	Random forest lag	1	Average NIBUT	8	0.416	0.339	4.488	4.350	0.276	0.815
Eye-level primary	Random forest lag	1	First NIBUT	8	0.305	0.248	4.390	4.265	0.184	0.805
Eye-level primary	Random forest lag	1	TMH	8	0.015	0.011	5.616	5.371	0.008	0.947
Eye-level primary	Random forest lag	2	Average NIBUT	8	0.319	0.243	3.195	3.107	0.227	0.624
Eye-level primary	Random forest lag	2	First NIBUT	8	0.363	0.276	4.902	4.717	0.179	0.958
Eye-level primary	Random forest lag	2	TMH	8	0.009	0.006	2.944	2.885	0.002	0.574
Eye-level primary	Random forest lag	3	Average NIBUT	8	0.969	0.716	10.654	9.612	0.658	1.896
Eye-level primary	Random forest lag	3	First NIBUT	8	0.623	0.510	9.555	8.920	0.456	1.643
Eye-level primary	Random forest lag	3	TMH	8	0.030	0.018	7.647	8.054	−0.007	1.922
Eye-level primary	SARIMA(1,0,0)(1,0,0,12)	1	Average NIBUT	8	0.301	0.259	3.395	3.325	0.204	0.589
Eye-level primary	SARIMA(1,0,0)(1,0,0,12)	1	First NIBUT	8	0.223	0.185	3.259	3.185	0.171	0.587
Eye-level primary	SARIMA(1,0,0)(1,0,0,12)	1	TMH	8	0.019	0.011	5.837	5.437	0.008	1.216
Eye-level primary	SARIMA(1,0,0)(1,0,0,12)	2	Average NIBUT	8	0.311	0.253	3.315	3.233	0.222	0.608
Eye-level primary	SARIMA(1,0,0)(1,0,0,12)	2	First NIBUT	8	0.296	0.245	4.320	4.201	0.148	0.780
Eye-level primary	SARIMA(1,0,0)(1,0,0,12)	2	TMH	8	0.011	0.008	3.880	3.851	−0.001	0.681
Eye-level primary	SARIMA(1,0,0)(1,0,0,12)	3	Average NIBUT	8	0.958	0.697	10.403	9.376	0.635	1.875
Eye-level primary	SARIMA(1,0,0)(1,0,0,12)	3	First NIBUT	8	0.549	0.458	8.583	8.079	0.389	1.447
Eye-level primary	SARIMA(1,0,0)(1,0,0,12)	3	TMH	8	0.027	0.017	6.918	7.402	−0.011	1.735
Eye-level primary	Seasonal naive	1	Average NIBUT	8	0.610	0.548	7.206	6.931	0.388	1.193
Eye-level primary	Seasonal naive	1	First NIBUT	8	0.421	0.373	6.549	6.356	0.209	1.110
Eye-level primary	Seasonal naive	1	TMH	8	0.008	0.005	2.471	2.416	0.003	0.497
Eye-level primary	Seasonal naive	2	Average NIBUT	8	0.511	0.432	5.609	5.520	0.217	1.000
Eye-level primary	Seasonal naive	2	First NIBUT	8	0.448	0.407	7.112	6.998	0.145	1.182
Eye-level primary	Seasonal naive	2	TMH	8	0.011	0.009	4.091	4.122	0.002	0.695
Eye-level primary	Seasonal naive	3	Average NIBUT	8	0.996	0.734	10.889	9.799	0.686	1.949

Dataset	Model	Horizon	Target	Forecasts	RMSE	MAE	MAPE	sMAPE	Bias	Normalized RMSE
Eye-level primary	Seasonal naive	3	First NIBUT	8	0.548	0.456	8.485	7.980	0.456	1.445
Eye-level primary	Seasonal naive	3	TMH	8	0.029	0.018	7.366	7.971	−0.011	1.895
Eye-level primary	Simple exponential smoothing	1	Average NIBUT	8	0.372	0.254	3.361	3.405	−0.038	0.729
Eye-level primary	Simple exponential smoothing	1	First NIBUT	8	0.216	0.166	2.928	2.882	0.041	0.571
Eye-level primary	Simple exponential smoothing	1	TMH	8	0.022	0.013	6.635	6.084	0.010	1.448
Eye-level primary	Simple exponential smoothing	2	Average NIBUT	8	0.374	0.259	3.374	3.490	−0.162	0.733
Eye-level primary	Simple exponential smoothing	2	First NIBUT	8	0.281	0.225	3.836	3.878	−0.045	0.740
Eye-level primary	Simple exponential smoothing	2	TMH	8	0.020	0.015	7.400	7.107	0.003	1.309
Eye-level primary	Simple exponential smoothing	3	Average NIBUT	8	0.844	0.595	8.880	8.233	0.218	1.652
Eye-level primary	Simple exponential smoothing	3	First NIBUT	8	0.425	0.295	5.566	5.280	0.194	1.121
Eye-level primary	Simple exponential smoothing	3	TMH	8	0.029	0.021	9.059	9.081	−0.006	1.846

B. Prediction-interval coverage

Model	Target	Forecast horizon	Number of forecast origins	95% PI coverage	Mean interval width	Median interval width
ARIMA(1,1,0)	Average NIBUT	1	8	1.000	1.962	1.986
ARIMA(1,1,0)	Average NIBUT	2	8	1.000	2.434	2.436
ARIMA(1,1,0)	Average NIBUT	3	8	0.875	2.905	2.902
ARIMA(1,1,0)	First NIBUT	1	8	1.000	1.504	1.495
ARIMA(1,1,0)	First NIBUT	2	8	1.000	1.849	1.843
ARIMA(1,1,0)	First NIBUT	3	8	1.000	2.203	2.196
ARIMA(1,1,0)	TMH	1	8	0.875	0.051	0.049
ARIMA(1,1,0)	TMH	2	8	0.875	0.062	0.062
ARIMA(1,1,0)	TMH	3	8	0.750	0.074	0.074
Linear trend	Average NIBUT	1	8	1.000	2.183	2.217
Linear trend	Average NIBUT	2	8	1.000	2.187	2.223
Linear trend	Average NIBUT	3	8	0.875	2.192	2.228
Linear trend	First NIBUT	1	8	1.000	1.540	1.536
Linear trend	First NIBUT	2	8	1.000	1.543	1.539
Linear trend	First NIBUT	3	8	0.875	1.547	1.541
Linear trend	TMH	1	8	1.000	0.058	0.058

Model	Target	Forecast horizon	Number of forecast origins	95% PI coverage	Mean interval width	Median interval width
Linear trend	TMH	2	8	1.000	0.059	0.058
Linear trend	TMH	3	8	0.750	0.059	0.058
SARIMA(1,0,0)(1,0,0,12)	Average NIBUT	1	8	1.000	1.814	1.835
SARIMA(1,0,0)(1,0,0,12)	Average NIBUT	2	8	1.000	1.937	1.968
SARIMA(1,0,0)(1,0,0,12)	Average NIBUT	3	8	0.875	1.957	1.984
SARIMA(1,0,0)(1,0,0,12)	First NIBUT	1	8	1.000	1.395	1.402
SARIMA(1,0,0)(1,0,0,12)	First NIBUT	2	8	1.000	1.468	1.490
SARIMA(1,0,0)(1,0,0,12)	First NIBUT	3	8	0.875	1.478	1.494
SARIMA(1,0,0)(1,0,0,12)	TMH	1	8	0.875	0.047	0.047
SARIMA(1,0,0)(1,0,0,12)	TMH	2	8	1.000	0.049	0.048
SARIMA(1,0,0)(1,0,0,12)	TMH	3	8	0.750	0.049	0.049

Table S6. Robustness analyses: aggregation, sample-size weighting, and pandemic-period sensitivity

A. Eye-level and patient-month benchmark results

Dataset	Model	Macro nRMSE	Mean rank	Overall rank	Eye- vs patient-month rank correlation	Rank-correlation P
Eye-level primary	ARIMA(1,1,0)	1.003	4.875	4	1.000	<0.001
Eye-level primary	Last-value naive	1.100	4.875	7	1.000	<0.001
Eye-level primary	Linear trend	0.875	4.125	1	1.000	<0.001
Eye-level primary	Random forest lag	1.016	5.500	5	1.000	<0.001
Eye-level primary	SARIMA(1,0,0)(1,0,0,12)	0.940	4.000	3	1.000	<0.001
Eye-level primary	Seasonal naive	1.099	7.250	6	1.000	<0.001
Eye-level primary	Simple exponential smoothing	0.907	3.500	2	1.000	<0.001
Patient-month	ARIMA(1,1,0)	0.957	3.875	4	1.000	<0.001
Patient-month	Last-value naive	1.037	4.250	7	1.000	<0.001
Patient-month	Linear trend	0.828	3.625	1	1.000	<0.001
Patient-month	Random forest lag	0.958	3.875	5	1.000	<0.001
Patient-month	SARIMA(1,0,0)(1,0,0,12)	0.890	3.625	3	1.000	<0.001
Patient-month	Seasonal naive	1.029	6.000	6	1.000	<0.001
Patient-month	Simple exponential smoothing	0.868	2.750	2	1.000	<0.001

B. Sample-size-weighted model summary

Model	Mean macro normalized weighted RMSE
ARIMA(1,1,0)	1.150
Last-value naive	1.286
Linear trend	0.943
Random forest lag	1.163
SARIMA(1,0,0)(1,0,0,12)	1.052
Seasonal naive	1.298
Simple exponential smoothing	1.006

C. Exploratory pandemic-period segmented analysis

Outcome	Term	Coefficient	Robust SE	P value	CI lower	CI upper	Interpretation
Monthly eye-level volume	Intercept	458.636	127.215	<0.001	209.300	707.973	Descriptive only; not intended for causal inference
Monthly eye-level volume	Time	−2.978	11.785	0.801	−26.076	20.120	Descriptive only; not intended for causal inference
Monthly eye-level volume	Post-disruption level	155.512	148.974	0.297	−136.471	447.495	Descriptive only; not intended for causal inference
Monthly eye-level volume	Post-disruption slope	1.091	11.971	0.927	−22.373	24.555	Descriptive only; not intended for causal inference
First NIBUT	Intercept	5.989	0.268	<0.001	5.464	6.513	Descriptive only; not intended for causal inference
First NIBUT	Time	0.007	0.031	0.823	−0.054	0.068	Descriptive only; not intended for causal inference
First NIBUT	Post-disruption level	−0.024	0.428	0.955	−0.863	0.816	Descriptive only; not intended for causal inference
First NIBUT	Post-disruption slope	−0.019	0.031	0.541	−0.080	0.042	Descriptive only; not intended for causal inference

Outcome	Term	Coefficient	Robust SE	P value	CI lower	CI upper	Interpretation
Average NIBUT	Intercept	8.125	0.427	<0.001	7.289	8.962	Descriptive only; not intended for causal inference
Average NIBUT	Time	0.004	0.041	0.919	−0.077	0.085	Descriptive only; not intended for causal inference
Average NIBUT	Post-disruption level	0.149	0.512	0.771	−0.855	1.152	Descriptive only; not intended for causal inference
Average NIBUT	Post-disruption slope	−0.026	0.042	0.539	−0.107	0.056	Descriptive only; not intended for causal inference
TMH	Intercept	0.235	0.007	<0.001	0.222	0.248	Descriptive only; not intended for causal inference
TMH	Time	−0.001	0.001	0.091	−0.002	0.000	Descriptive only; not intended for causal inference
TMH	Post-disruption level	−0.007	0.009	0.416	−0.026	0.011	Descriptive only; not intended for causal inference
TMH	Post-disruption slope	0.001	0.001	0.057	−0.000	0.003	Descriptive only; not intended for causal inference

Table S7. TMH missingness analysis

A. Monthly missingness

Calendar month	Total	TMH missing	TMH available	Unique patients	Monthly eye-level volume	TMH missing (%)
2018-07	81	59	22	40	81	72.84
2018-08	113	94	19	52	113	83.19
2018-09	376	43	333	183	376	11.44
2018-10	399	39	360	197	399	9.77
2018-11	643	39	604	313	643	6.07
2018-12	643	79	564	303	643	12.29
2019-01	515	42	473	245	515	8.16
2019-02	368	29	339	183	368	7.88

Calendar month	Total	TMH missing	TMH available	Unique patients	Monthly eye-level volume	TMH missing (%)
2019-03	716	74	642	350	716	10.34
2019-04	660	51	609	312	660	7.73
2019-05	524	22	502	249	524	4.20
2019-06	425	17	408	209	425	4.00
2019-07	560	17	543	275	560	3.04
2019-08	512	14	498	248	512	2.73
2019-09	489	8	481	235	489	1.64
2019-10	526	15	511	261	526	2.85
2019-11	574	11	563	279	574	1.92
2019-12	507	20	487	233	507	3.94
2020-01	371	11	360	171	371	2.96
2020-02	4	0	4	2	4	0.00
2020-04	299	8	291	143	299	2.68
2020-05	491	20	471	239	491	4.07
2020-06	470	16	454	222	470	3.40
2020-07	302	6	296	150	302	1.99
2020-08	551	15	536	271	551	2.72
2020-09	737	65	672	310	737	8.82
2020-10	502	270	232	245	502	53.78
2020-11	581	354	227	278	581	60.93
2020-12	470	242	228	231	470	51.49
2021-01	470	272	198	229	470	57.87
2021-02	360	220	140	178	360	61.11
2021-03	622	347	275	296	622	55.79
2021-04	573	326	247	275	573	56.89
2021-05	539	284	255	253	539	52.69
2021-06	575	299	276	251	575	52.00
2021-07	429	283	146	202	429	65.97
2021-08	411	245	166	198	411	59.61

Calendar month	Total	TMH missing	TMH available	Unique patients	Monthly eye-level volume	TMH missing (%)
2021-09	595	386	209	267	595	64.87
2021-10	518	299	219	230	518	57.72
2021-11	715	419	296	322	715	58.60
2021-12	871	442	429	392	871	50.75
2022-01	702	381	321	334	702	54.27
2022-02	663	363	300	322	663	54.75
2022-03	861	338	523	417	861	39.26
2022-04	525	274	251	259	525	52.19
2022-05	363	231	132	175	363	63.64
2022-06	509	305	204	242	509	59.92
2022-07	547	197	350	263	547	36.01
2022-08	739	216	523	320	739	29.23
2022-09	629	199	430	288	629	31.64
2022-10	484	47	437	241	484	9.71
2022-11	470	59	411	233	470	12.55
2022-12	32	8	24	17	32	25.00
2023-01	315	42	273	156	315	13.33
2023-02	570	76	494	286	570	13.33
2023-03	627	50	577	309	627	7.97
2023-04	591	52	539	292	591	8.80
2023-05	452	57	395	223	452	12.61
2023-06	357	30	327	180	357	8.40
2023-07	489	26	463	243	489	5.32
2023-08	534	33	501	267	534	6.18
2023-09	438	32	406	218	438	7.31
2023-10	438	21	417	218	438	4.79
2023-11	70	3	67	35	70	4.29

B. Yearly missingness

Year	Total	TMH missing	TMH available	TMH missing (%)

Year	Total	TMH missing	TMH available	TMH missing (%)
2018	2,255	353	1,902	15.65
2019	6,376	320	6,056	5.02
2020	4,778	1,007	3,771	21.08
2021	6,678	3,822	2,856	57.23
2022	6,524	2,618	3,906	40.13
2023	4,881	422	4,459	8.65

C. Missingness by age stratum and laterality

Stratum	Total	TMH missing	Missing (%)	Characteristic
18–35 years	8,945	2,607	29.14	Age stratum
36–55 years	11,903	3,193	26.83	Age stratum
≥56 years	10,644	2,742	25.76	Age stratum
Left	15,724	4,290	27.28	Laterality
Right	15,768	4,252	26.97	Laterality

D. Association with monthly examination volume

Method	Correlation coefficient	P value	Months
Pearson	0.061	0.633	64
Spearman	0.131	0.303	64

Table S8. Age-stratified data composition and model performance

A. Age-stratified model performance

Age stratum	Model	Macro nRMSE	Mean rank	Overall rank
18–35 years	ARIMA(1,1,0)	0.910	3.125	3
18–35 years	Last-value naive	1.023	4.375	5
18–35 years	Linear trend	0.901	3.750	2
18–35 years	Random forest lag	1.163	4.875	7
18–35 years	SARIMA(1,0,0)(1,0,0,12)	0.938	4.125	4
18–35 years	Seasonal naive	1.082	5.375	6
18–35 years	Simple exponential smoothing	0.884	2.375	1
36–55 years	ARIMA(1,1,0)	1.115	3.875	5

Age stratum	Model	Macro nRMSE	Mean rank	Overall rank
36–55 years	Last-value naive	1.158	3.875	6
36–55 years	Linear trend	0.979	4.000	2
36–55 years	Random forest lag	1.074	5.000	4
36–55 years	SARIMA(1,0,0)(1,0,0,12)	0.914	2.250	1
36–55 years	Seasonal naive	1.207	5.375	7
36–55 years	Simple exponential smoothing	0.987	3.625	3
≥56 years	ARIMA(1,1,0)	1.162	4.250	5
≥56 years	Last-value naive	1.290	5.000	6
≥56 years	Linear trend	0.928	2.750	1
≥56 years	Random forest lag	1.057	3.750	4
≥56 years	SARIMA(1,0,0)(1,0,0,12)	1.039	3.250	3
≥56 years	Seasonal naive	1.333	6.000	7
≥56 years	Simple exponential smoothing	1.034	3.000	2

B. Age-stratified monthly composition: 18–35 years

Calendar month	Age stratum	Eye-level records	Unique examinations	Unique patients	Examinations per patient	Eye records per patient	First NIBUT available	Average NIBUT available	TMH available	TMH missing	TMH missing (%)
2018-07	18–35 years	23	11	11	1.000	2.091	23	23	8	15	65.22
2018-08	18–35 years	30	15	15	1.000	2.000	30	30	8	22	73.33
2018-09	18–35 years	67	34	33	1.030	2.030	67	67	62	5	7.46
2018-10	18–35 years	106	53	52	1.019	2.038	106	106	97	9	8.49
2018-11	18–35 years	120	60	59	1.017	2.034	120	120	110	10	8.33
2018-12	18–35 years	187	93	91	1.022	2.055	185	185	161	26	13.90
2019-01	18–35 years	140	67	67	1.000	2.090	140	140	130	10	7.14
2019-02	18–35 years	57	29	29	1.000	1.966	57	57	53	4	7.02
2019-03	18–35 years	153	76	75	1.013	2.040	152	152	144	9	5.88
2019-04	18–35 years	140	70	65	1.077	2.154	140	140	132	8	5.71
2019-05	18–35 years	122	59	58	1.017	2.103	122	122	118	4	3.28
2019-06	18–35 years	99	50	50	1.000	1.980	99	99	96	3	3.03
2019-07	18–35 years	140	70	69	1.014	2.029	140	140	140	0	0.00

Calendar month	Age stratum	Eye-level records	Unique examinations	Unique patients	Examinations per patient	Eye records per patient	First NIBUT available	Average NIBUT available	TMH available	TMH missing	TMH missing (%)
2019-08	18–35 years	97	47	46	1.022	2.109	97	97	94	3	3.09
2019-09	18–35 years	64	32	32	1.000	2.000	64	64	64	0	0.00
2019-10	18–35 years	105	53	52	1.019	2.019	105	105	103	2	1.90
2019-11	18–35 years	132	63	61	1.033	2.164	132	132	132	0	0.00
2019-12	18–35 years	87	41	41	1.000	2.122	87	87	86	1	1.15
2020-01	18–35 years	98	45	45	1.000	2.178	98	98	96	2	2.04
2020-02	18–35 years	2	1	1	1.000	2.000	2	2	2	0	0.00
2020-03	18–35 years	0	0	0			0	0	0	0	
2020-04	18–35 years	116	56	56	1.000	2.071	116	116	113	3	2.59
2020-05	18–35 years	139	69	69	1.000	2.014	139	139	137	2	1.44
2020-06	18–35 years	98	47	46	1.022	2.130	98	98	95	3	3.06
2020-07	18–35 years	95	47	47	1.000	2.021	95	95	93	2	2.11
2020-08	18–35 years	139	68	68	1.000	2.044	139	139	133	6	4.32
2020-09	18–35 years	106	53	53	1.000	2.000	106	106	93	13	12.26
2020-10	18–35 years	97	47	47	1.000	2.064	97	97	46	51	52.58
2020-11	18–35 years	142	66	66	1.000	2.152	142	142	48	94	66.20
2020-12	18–35 years	146	71	71	1.000	2.056	146	146	85	61	41.78
2021-01	18–35 years	160	78	78	1.000	2.051	160	160	66	94	58.75
2021-02	18–35 years	93	46	46	1.000	2.022	93	93	38	55	59.14
2021-03	18–35 years	178	86	83	1.036	2.145	178	178	62	116	65.17
2021-04	18–35 years	153	74	74	1.000	2.068	153	153	76	77	50.33
2021-05	18–35 years	146	69	68	1.015	2.147	146	146	65	81	55.48
2021-06	18–35 years	144	68	64	1.062	2.250	144	144	67	77	53.47
2021-07	18–35 years	121	57	55	1.036	2.200	121	121	34	87	71.90
2021-08	18–35 years	144	71	71	1.000	2.028	144	144	59	85	59.03
2021-09	18–35 years	144	64	64	1.000	2.250	144	144	55	89	61.81
2021-10	18–35 years	144	64	64	1.000	2.250	144	144	75	69	47.92
2021-11	18–35 years	261	116	116	1.000	2.250	261	261	107	154	59.00
2021-12	18–35 years	309	141	139	1.014	2.223	309	309	140	169	54.69

Calendar month	Age stratum	Eye-level records	Unique examinations	Unique patients	Examinations per patient	Eye records per patient	First NIBUT available	Average NIBUT available	TMH available	TMH missing	TMH missing (%)
2022-01	18–35 years	272	129	128	1.008	2.125	272	272	112	160	58.82
2022-02	18–35 years	286	139	139	1.000	2.058	286	286	126	160	55.94
2022-03	18–35 years	341	166	165	1.006	2.067	341	341	208	133	39.00
2022-04	18–35 years	215	106	105	1.010	2.048	215	215	107	108	50.23
2022-05	18–35 years	166	80	79	1.013	2.101	166	166	63	103	62.05
2022-06	18–35 years	199	96	95	1.011	2.095	198	198	80	119	59.80
2022-07	18–35 years	189	93	93	1.000	2.032	189	189	136	53	28.04
2022-08	18–35 years	215	107	103	1.039	2.087	215	215	182	33	15.35
2022-09	18–35 years	182	89	88	1.011	2.068	182	182	142	40	21.98
2022-10	18–35 years	166	84	84	1.000	1.976	166	166	144	22	13.25
2022-11	18–35 years	181	92	92	1.000	1.967	181	181	164	17	9.39
2022-12	18–35 years	10	6	6	1.000	1.667	10	10	6	4	40.00
2023-01	18–35 years	124	62	62	1.000	2.000	124	124	107	17	13.71
2023-02	18–35 years	200	101	101	1.000	1.980	200	200	176	24	12.00
2023-03	18–35 years	208	105	101	1.040	2.059	208	208	179	29	13.94
2023-04	18–35 years	238	118	117	1.009	2.034	237	237	220	18	7.56
2023-05	18–35 years	98	48	48	1.000	2.042	98	98	82	16	16.33
2023-06	18–35 years	110	55	55	1.000	2.000	110	110	99	11	10.00
2023-07	18–35 years	148	74	74	1.000	2.000	148	148	142	6	4.05
2023-08	18–35 years	122	61	61	1.000	2.000	122	122	115	7	5.74
2023-09	18–35 years	93	44	44	1.000	2.114	93	93	90	3	3.23
2023-10	18–35 years	130	64	64	1.000	2.031	130	130	127	3	2.31
2023-11	18–35 years	8	4	4	1.000	2.000	8	8	8	0	0.00

C. Age-stratified monthly composition: 36–55 years

Calendar month	Age stratum	Eye-level records	Unique examinations	Unique patients	Examinations per patient	Eye records per patient	First NIBUT available	Average NIBUT available	TMH available	TMH missing	TMH missing (%)
2018-07	36–55 years	24	12	12	1.000	2.000	24	24	4	20	83.33
2018-08	36–55 years	44	22	22	1.000	2.000	44	44	8	36	81.82
2018-09	36–55 years	130	64	63	1.016	2.063	130	130	120	10	7.69

Calendar month	Age stratum	Eye-level records	Unique examinations	Unique patients	Examinations per patient	Eye records per patient	First NIBUT available	Average NIBUT available	TMH available	TMH missing	TMH missing (%)
2018-10	36–55 years	158	79	78	1.013	2.026	158	158	141	17	10.76
2018-11	36–55 years	251	124	123	1.008	2.041	251	251	235	16	6.37
2018-12	36–55 years	249	121	117	1.034	2.128	249	249	226	23	9.24
2019-01	36–55 years	229	114	112	1.018	2.045	228	228	209	20	8.73
2019-02	36–55 years	145	73	72	1.014	2.014	145	145	138	7	4.83
2019-03	36–55 years	251	124	122	1.016	2.057	251	251	223	28	11.16
2019-04	36–55 years	269	134	128	1.047	2.102	269	269	246	23	8.55
2019-05	36–55 years	210	103	102	1.010	2.059	210	210	203	7	3.33
2019-06	36–55 years	163	79	79	1.000	2.063	163	163	154	9	5.52
2019-07	36–55 years	200	98	97	1.010	2.062	199	199	196	4	2.00
2019-08	36–55 years	168	83	81	1.025	2.074	168	168	165	3	1.79
2019-09	36–55 years	154	74	74	1.000	2.081	153	153	152	2	1.30
2019-10	36–55 years	182	91	91	1.000	2.000	182	182	178	4	2.20
2019-11	36–55 years	206	102	102	1.000	2.020	206	206	201	5	2.43
2019-12	36–55 years	187	86	85	1.012	2.200	186	186	182	5	2.67
2020-01	36–55 years	138	65	65	1.000	2.123	138	138	133	5	3.62
2020-02	36–55 years	2	1	1	1.000	2.000	2	2	2	0	0.00
2020-03	36–55 years	0	0	0			0	0	0	0	
2020-04	36–55 years	96	46	46	1.000	2.087	96	96	94	2	2.08
2020-05	36–55 years	193	94	94	1.000	2.053	192	192	188	5	2.59
2020-06	36–55 years	201	97	97	1.000	2.072	201	201	199	2	1.00
2020-07	36–55 years	99	49	49	1.000	2.020	98	98	98	1	1.01
2020-08	36–55 years	188	91	91	1.000	2.066	188	188	185	3	1.60
2020-09	36–55 years	248	122	111	1.099	2.234	246	246	233	15	6.05
2020-10	36–55 years	164	81	80	1.012	2.050	164	164	70	94	57.32
2020-11	36–55 years	242	117	117	1.000	2.068	242	242	102	140	57.85
2020-12	36–55 years	189	93	93	1.000	2.032	189	189	83	106	56.08
2021-01	36–55 years	180	89	88	1.011	2.045	180	180	78	102	56.67
2021-02	36–55 years	149	73	73	1.000	2.041	149	149	49	100	67.11

Calendar month	Age stratum	Eye-level records	Unique examinations	Unique patients	Examinations per patient	Eye records per patient	First NIBUT available	Average NIBUT available	TMH available	TMH missing	TMH missing (%)
2021-03	36–55 years	238	115	115	1.000	2.070	238	238	125	113	47.48
2021-04	36–55 years	251	120	119	1.008	2.109	251	251	106	145	57.77
2021-05	36–55 years	205	95	94	1.011	2.181	205	205	103	102	49.76
2021-06	36–55 years	237	114	109	1.046	2.174	237	237	110	127	53.59
2021-07	36–55 years	152	73	73	1.000	2.082	152	152	58	94	61.84
2021-08	36–55 years	151	70	70	1.000	2.157	151	151	54	97	64.24
2021-09	36–55 years	245	108	107	1.009	2.290	245	245	83	162	66.12
2021-10	36–55 years	222	98	97	1.010	2.289	222	222	88	134	60.36
2021-11	36–55 years	272	124	124	1.000	2.194	271	271	99	173	63.60
2021-12	36–55 years	357	162	162	1.000	2.204	357	357	186	171	47.90
2022-01	36–55 years	253	121	121	1.000	2.091	253	253	131	122	48.22
2022-02	36–55 years	246	119	119	1.000	2.067	246	246	119	127	51.63
2022-03	36–55 years	302	148	144	1.028	2.097	302	302	178	124	41.06
2022-04	36–55 years	184	91	91	1.000	2.022	184	184	78	106	57.61
2022-05	36–55 years	107	53	53	1.000	2.019	107	107	43	64	59.81
2022-06	36–55 years	199	97	96	1.010	2.073	199	199	81	118	59.30
2022-07	36–55 years	194	96	95	1.011	2.042	194	194	121	73	37.63
2022-08	36–55 years	263	128	117	1.094	2.248	263	263	202	61	23.19
2022-09	36–55 years	223	110	103	1.068	2.165	223	223	155	68	30.49
2022-10	36–55 years	187	92	92	1.000	2.033	187	187	170	17	9.09
2022-11	36–55 years	189	94	94	1.000	2.011	189	189	168	21	11.11
2022-12	36–55 years	14	7	7	1.000	2.000	14	14	12	2	14.29
2023-01	36–55 years	120	60	58	1.034	2.069	119	119	106	14	11.67
2023-02	36–55 years	236	118	118	1.000	2.000	236	236	206	30	12.71
2023-03	36–55 years	238	119	117	1.017	2.034	238	238	223	15	6.30
2023-04	36–55 years	194	97	96	1.010	2.021	194	194	176	18	9.28
2023-05	36–55 years	190	95	94	1.011	2.021	190	190	169	21	11.05
2023-06	36–55 years	155	79	79	1.000	1.962	155	155	145	10	6.45
2023-07	36–55 years	189	94	93	1.011	2.032	189	189	177	12	6.35

Calendar month	Age stratum	Eye-level records	Unique examinations	Unique patients	Examinations per patient	Eye records per patient	First NIBUT available	Average NIBUT available	TMH available	TMH missing	TMH missing (%)
2023-08	36–55 years	222	111	111	1.000	2.000	222	222	212	10	4.50
2023-09	36–55 years	157	79	79	1.000	1.987	157	157	141	16	10.19
2023-10	36–55 years	162	81	81	1.000	2.000	161	161	153	9	5.56
2023-11	36–55 years	40	20	20	1.000	2.000	40	40	37	3	7.50

D. Age-stratified monthly composition: ≥56 years

Calendar month	Age stratum	Eye-level records	Unique examinations	Unique patients	Examinations per patient	Eye records per patient	First NIBUT available	Average NIBUT available	TMH available	TMH missing	TMH missing (%)
2018-07	≥56 years	34	17	17	1.000	2.000	34	34	10	24	70.59
2018-08	≥56 years	39	16	15	1.067	2.600	39	39	3	36	92.31
2018-09	≥56 years	179	87	87	1.000	2.057	179	179	151	28	15.64
2018-10	≥56 years	135	67	67	1.000	2.015	135	135	122	13	9.63
2018-11	≥56 years	272	133	131	1.015	2.076	272	272	259	13	4.78
2018-12	≥56 years	207	101	95	1.063	2.179	207	207	177	30	14.49
2019-01	≥56 years	146	72	66	1.091	2.212	146	146	134	12	8.22
2019-02	≥56 years	166	82	82	1.000	2.024	166	166	148	18	10.84
2019-03	≥56 years	312	155	153	1.013	2.039	311	311	275	37	11.86
2019-04	≥56 years	251	125	119	1.050	2.109	250	250	231	20	7.97
2019-05	≥56 years	192	93	89	1.045	2.157	192	192	181	11	5.73
2019-06	≥56 years	163	80	80	1.000	2.038	163	163	158	5	3.07
2019-07	≥56 years	220	109	109	1.000	2.018	219	219	207	13	5.91
2019-08	≥56 years	247	123	121	1.017	2.041	247	247	239	8	3.24
2019-09	≥56 years	271	129	129	1.000	2.101	270	270	265	6	2.21
2019-10	≥56 years	239	118	118	1.000	2.025	239	239	230	9	3.77
2019-11	≥56 years	236	116	116	1.000	2.034	235	235	230	6	2.54
2019-12	≥56 years	233	108	107	1.009	2.178	232	232	219	14	6.01
2020-01	≥56 years	135	64	61	1.049	2.213	135	135	131	4	2.96
2020-02	≥56 years	0	0	0			0	0	0	0	
2020-03	≥56 years	0	0	0			0	0	0	0	

Calendar month	Age stratum	Eye-level records	Unique examinations	Unique patients	Examinations per patient	Eye records per patient	First NIBUT available	Average NIBUT available	TMH available	TMH missing	TMH missing (%)
2020-04	≥56 years	87	41	41	1.000	2.122	87	87	84	3	3.45
2020-05	≥56 years	159	76	76	1.000	2.092	159	159	146	13	8.18
2020-06	≥56 years	171	79	79	1.000	2.165	171	171	160	11	6.43
2020-07	≥56 years	108	54	54	1.000	2.000	108	108	105	3	2.78
2020-08	≥56 years	224	112	112	1.000	2.000	224	224	218	6	2.68
2020-09	≥56 years	383	194	146	1.329	2.623	382	382	346	37	9.66
2020-10	≥56 years	241	118	118	1.000	2.042	241	241	116	125	51.87
2020-11	≥56 years	197	95	95	1.000	2.074	197	197	77	120	60.91
2020-12	≥56 years	135	67	67	1.000	2.015	135	135	60	75	55.56
2021-01	≥56 years	130	63	63	1.000	2.063	130	130	54	76	58.46
2021-02	≥56 years	118	59	59	1.000	2.000	118	118	53	65	55.08
2021-03	≥56 years	206	99	98	1.010	2.102	206	206	88	118	57.28
2021-04	≥56 years	169	83	82	1.012	2.061	169	169	65	104	61.54
2021-05	≥56 years	188	91	91	1.000	2.066	188	188	87	101	53.72
2021-06	≥56 years	194	93	78	1.192	2.487	194	194	99	95	48.97
2021-07	≥56 years	156	78	74	1.054	2.108	156	156	54	102	65.38
2021-08	≥56 years	116	57	57	1.000	2.035	116	116	53	63	54.31
2021-09	≥56 years	206	97	96	1.010	2.146	206	206	71	135	65.53
2021-10	≥56 years	152	69	69	1.000	2.203	152	152	56	96	63.16
2021-11	≥56 years	182	82	82	1.000	2.220	182	182	90	92	50.55
2021-12	≥56 years	205	92	91	1.011	2.253	205	205	103	102	49.76
2022-01	≥56 years	177	85	85	1.000	2.082	177	177	78	99	55.93
2022-02	≥56 years	131	64	64	1.000	2.047	131	131	55	76	58.02
2022-03	≥56 years	218	108	108	1.000	2.019	218	218	137	81	37.16
2022-04	≥56 years	126	63	63	1.000	2.000	126	126	66	60	47.62
2022-05	≥56 years	90	44	43	1.023	2.093	90	90	26	64	71.11
2022-06	≥56 years	111	54	51	1.059	2.176	111	111	43	68	61.26
2022-07	≥56 years	164	79	75	1.053	2.187	164	164	93	71	43.29
2022-08	≥56 years	261	125	100	1.250	2.610	261	261	139	122	46.74

Calendar month	Age stratum	Eye-level records	Unique examinations	Unique patients	Examinations per patient	Eye records per patient	First NIBUT available	Average NIBUT available	TMH available	TMH missing	TMH missing (%)
2022-09	≥56 years	224	110	97	1.134	2.309	224	224	133	91	40.62
2022-10	≥56 years	131	66	65	1.015	2.015	131	131	123	8	6.11
2022-11	≥56 years	100	48	47	1.021	2.128	100	100	79	21	21.00
2022-12	≥56 years	8	4	4	1.000	2.000	8	8	6	2	25.00
2023-01	≥56 years	71	36	36	1.000	1.972	71	71	60	11	15.49
2023-02	≥56 years	134	67	67	1.000	2.000	134	134	112	22	16.42
2023-03	≥56 years	181	91	91	1.000	1.989	181	181	175	6	3.31
2023-04	≥56 years	159	79	79	1.000	2.013	159	159	143	16	10.06
2023-05	≥56 years	164	82	81	1.012	2.025	164	164	144	20	12.20
2023-06	≥56 years	92	46	46	1.000	2.000	92	92	83	9	9.78
2023-07	≥56 years	152	76	76	1.000	2.000	151	151	144	8	5.26
2023-08	≥56 years	190	95	95	1.000	2.000	190	190	174	16	8.42
2023-09	≥56 years	188	95	95	1.000	1.979	188	188	175	13	6.91
2023-10	≥56 years	146	73	73	1.000	2.000	145	145	137	9	6.16
2023-11	≥56 years	22	11	11	1.000	2.000	22	22	22	0	0.00
